# Clinical subtypes identification and feature recognition of sepsis leukocyte trajectories based on machine learning

**DOI:** 10.1038/s41598-025-96718-9

**Published:** 2025-04-10

**Authors:** ShengHui Miao, YiJing Liu, Min Li, Jing Yan

**Affiliations:** 1 The Fourth Affiliated Hospital, International Institutes of Medicine, Zhejiang University School of Medicine, Yiwu, 322000 China; 2 Department of Second Clinical Medical College, Zhejiang Chinese Medicine University, Hangzhou, 310053 Zhejiang China; 3 Zhejiang Hospital, Zhejiang University School of Medicine, Lingyin Road 12, Hangzhou, 310013 Zhejiang China

**Keywords:** Sepsis, Clinical subtypes, Leukocyte trajectories, Machine learning, Latent class analysis, Biomarkers, Infection

## Abstract

Sepsis is a highly variable condition, and tracking leukocyte patterns may offer insights for tailored treatment and prognosis. We used the MIMIC-IV database to analyze patients diagnosed with Sepsis-3 within 24 h of ICU admission. Latent class mixed models (LCMM) were applied to leukocyte trajectories to identify sepsis subtypes. The primary outcome was 28-day all-cause mortality, with secondary outcomes including the need for life-support therapies. Associations between leukocyte trajectories and outcomes were assessed using multivariate regression, and findings were externally validated with the eICU database. Use the XGBoost model to identify baseline characteristics of high-risk mortality sepsis subgroups for predicting subgroup allocation upon patient admission to the ICU, and apply the SHAP method to interpret the contributing variables of the model. Among 7410 sepsis patients, eight distinct leukocyte trajectory subtypes were identified. Among those subtypes, patients with persistently high leukocyte levels had the poorest prognosis (HR 3.00; 95% CI 2.48–3.62) and a significantly greater need for life-support therapies; Patients with persistently low white blood cell levels had a higher risk of death (HR 1.68; 95% CI 1.24–2.27) but were less likely to receive invasive mechanical ventilation. Incorporating early ICU baseline variables into an XGBoost algorithm enables effective prediction of high-mortality risk subgroups (AUC > 0.8). SHAP method reveals distinct early clinical characteristics between hyperinflammatory subtypes (class 4, 7, and 8) and the hypoinflammatory subtype (class 1). In ICU-admitted sepsis patients, eight leukocyte trajectories are identified, which is the key independent predictors of prognosis, separating from single leukocyte measurements. High-mortality risk subgroups exhibit distinct clinical characteristics at ICU admission, providing valuable insights for their prediction and personalized early intervention.

**Supplementary Information:**

The online version contains supplementary material available at 10.1038/s41598-025-96718-9.

## Introduction

Sepsis is currently defined as life-threatening organ dysfunction resulting from a dysregulated host response to infection^1,2^. It represents a major global health and economic challenge, contributing to approximately 19.7% of global deaths^3,4^. However, sepsis is a highly heterogeneous condition, with patients exhibiting varied responses to the same treatments and different patterns of organ dysfunction, which significantly impacts prognosis. Identifying distinct clinical subtypes of sepsis is therefore essential for advancing personalized treatment approaches and improving prognostic accuracy^5,6^. Uncontrolled systemic inflammation is a central feature of sepsis and plays a critical role in the development of organ dysfunction. Consequently, assessing a patient’s inflammatory status is a key component in managing the disease. Research has shown that clinical subtypes of sepsis can be differentiated based on patients’ inflammatory responses^7–10^. One of the most commonly used markers of inflammation is leukocyte count. Tracking the trajectory of leukocyte levels over time may offer deeper insights into a patient’s treatment response compared to a single static measurement. This suggests that leukocyte trajectories could be valuable for identifying distinct sepsis subtypes and guiding more precise treatment strategies^11,12^. The objective of this study was to develop, evaluate and predict sepsis subtypes. The first goal was to determine whether distinct leukocyte trajectory-based subtypes in patients with sepsis can be identified through the electronic health records. The second goal was to understand whether those different subtypes are associated with the patterns of biomarkers and clinical outcomes. The third goal was to determine whether the high-risk mortality subtypes can be identified using patient baseline characteristics and early-stage clinical features upon ICU admission.

## Methods

*Data sources*: This study utilized data from two large public databases: Development Cohort: Data were obtained from the Medical Information Mart for Intensive Care IV (MIMIC-IV) database, version 3.0. This dataset includes de-identified electronic health records from 364,627 patients hospitalized at Beth Israel Deaconess Medical Center between 2008 and 2019^13^.Validation Cohort: The eICU Collaborative Research Database, a multi-center U.S. database, provided de-identified health data from over 200,000 ICU admissions occurring between 2014 and 2015^14^.

**Fig. 1:**
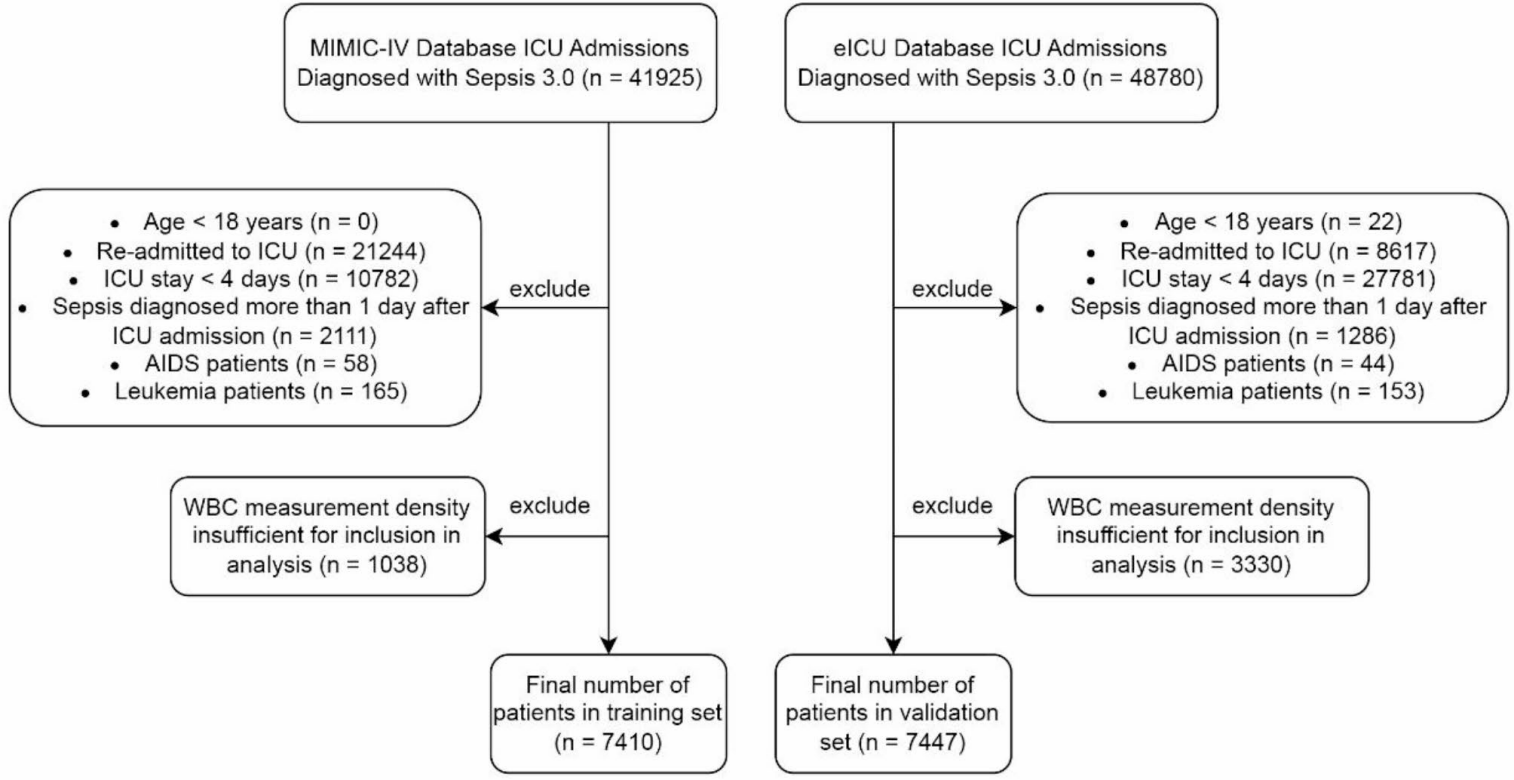
Flowchart illustrating the process of patient selection. Abbreviation: *MIMIC-IV* medical information mart for intensive care IV.

*Study population*: As illustrated in Fig. 1, this study included ICU patients diagnosed with sepsis based on Sepsis 3.0 criteria, defined as a suspected infection accompanied by an increase in the SOFA score of 2 points or more^1^. In the database, suspected infection was identified through the administration of intravenous antibiotics and the collection of blood cultures. The exclusion criteria were: (1) patients under 18 years of age; (2) patients with multiple ICU admissions; (3) ICU stays shorter than 4 days; (4) patients diagnosed with sepsis more than 24 h after ICU admission; (5) patients with AIDS; (6) patients with leukemia; and (7) patients lacking sufficient leukocyte count data for model construction (i.e., at least one leukocyte count recorded within each of the following time intervals: 0–24 h, 24–48 h, 48–72 h, and 72–96 h post-ICU admission).

*Outcomes*: Primary Outcome: 28-day all-cause mortality.Secondary Outcomes: The use of life-support treatments within 7 days, including vasopressors, invasive mechanical ventilation, and continuous renal replacement therapy (CRRT).

### Baseline characteristics

As detailed in Table 1, we collected demographic data (age, sex, ICU type, race, weight), SOFA scores, and information on chronic comorbidities (chronic heart failure, myocardial infarction, chronic lung disease, chronic kidney disease, chronic liver disease, rheumatologic disease, diabetes, and malignancy). Baseline vital signs (temperature, heart rate, respiratory rate, mean arterial pressure, and SpO_2_) and laboratory values (e.g., hemoglobin, leukocyte count, platelet count, albumin, liver enzymes, bicarbonate, blood urea nitrogen, creatinine, APTT, INR, pH, arterial blood gases, serum sodium, and potassium) were also collected.

**Table 1:** Baseline characteristics.

Database		MIMIC-IV (*N* = 7410)	eICU (*N* = 7447)	*p*
Class	1	182 (2.5%)	213 (2.9%)	< 0.001
	2	1382 (18.7%)	1320 (17.7%)	
	3	2710 (36.6%)	2176 (29.2%)	
	4	438 (5.9%)	571 (7.7%)	
	5	1158 (15.6%)	1354 (18.2%)	
	6	1168 (15.8%)	1201 (16.1%)	
	7	173 (2.3%)	277 (3.7%)	
	8	199 (2.7%)	335 (4.5%)	
Age		66.8 (55.0–77.6)	66.0 (54.0–76.0)	< 0.001
Gender	Male	4287 (57.9%)	3976 (53.4%)	< 0.001
	Female	3123 (42.1%)	3471 (46.6%)	
ICU type	SICU	3208 (43.3%)	5007 (67.2%)	< 0.001
	CCU	1834 (24.8%)	1281 (17.2%)	
	OTHER	2368 (32%)	1159 (15.6%)	
Race	WHITE	4602 (62.1%)	5715 (76.7%)	< 0.001
	ASIAN	192 (2.6%)	128 (1.7%)	
	BLACK	604 (8.2%)	802 (10.8%)	
	LATINO	221 (3%)	346 (4.6%)	
	OTHER	1791 (24.2%)	456 (6.1%)	
Weight		80.3 (67.8–97.9)	80.0 (65.7–99.0)	0.060
SOFA		8.0 (6.0–11.0)	8.0 (6.0–11.0)	< 0.001
Comorbidity	CHF	2498 (33.7%)	1713 (23%)	< 0.001
	MI	1379 (18.6%)	1261 (16.9%)	0.008
	CPD	2141 (28.9%)	2322 (31.2%)	0.003
	CKD	1683 (22.7%)	1620 (21.8%)	0.166
	Rheumatism	286 (3.9%)	221 (3%)	0.003
	Liver disease	1451 (19.6%)	590 (7.9%)	< 0.001
	Diabetes	2284 (30.8%)	2465 (33.1%)	0.003
	Cancer	879 (11.9%)	1152 (15.5%)	< 0.001
Vital signs	Temperature	37.9 (36.2–38.7)	37.9 (35.9–38.7)	0.201
	HR	120.0 (104.0–135.0)	125.0 (108.0–141.0)	< 0.001
	RR	33.0 (29.0–38.0)	34.0 (29.0–41.0)	< 0.001
	MAP	52.0 (45.0–58.0)	51.0 (44.0–59.0)	0.575
	SpO2	89.0 (85.0–92.0)	87.0 (81.0–91.0)	< 0.001
Laboratory	Hemoglobin	8.6 (7.5–10.0)	8.6 (7.4–10.1)	0.494
	WBC	15.9 (11.9–21.2)	17.5 (12.7–24.0)	< 0.001
	Plt	130.0 (80.0–192.0)	140.0 (85.0–200.0)	< 0.001
	Albumin	2.8 (2.3–3.2)	2.2 (1.8–2.7)	< 0.001
	ALT	35.0 (19.0–90.0)	35.0 (20.0–75.0)	0.261
	AST	57.0 (30.0–145.0)	47.0 (27.0–111.0)	< 0.001
	Bicarbonate	20.0 (17.0–29.0)	26.0 (17.0–31.0)	< 0.001
	Bun	33.0 (20.0–54.0)	39.0 (24.0–62.0)	< 0.001
	Cr	1.4 (0.9–2.6)	1.7 (1.0–3.1)	< 0.001
	Chloride	109.0 (97.0–113.0)	110.0 (97.0–115.0)	< 0.001
	APTT	38.2 (30.9–65.3)	36.0 (30.0–50.4)	< 0.001
	INR	1.4 (1.2–1.9)	1.4 (1.2–1.8)	< 0.001
	pH	7.3 (7.2–7.5)	7.3 (7.2–7.4)	< 0.001
	PaO2	73.0 (62.0–90.0)	65.2 (54.0–81.0)	< 0.001
	PaO2/FiO2	150.0 (95.0–226.7)	141.0 (84.4–220.0)	< 0.001
	PaCO2	47.0 (40.0–55.0)	47.0 (39.0–58.0)	0.729
	Sodium	135.0 (132.0–139.0)	136.0 (132.0–147.0)	< 0.001
	Potassium	3.6 (3.3–4.3)	3.3 (3.0–3.8)	< 0.001
	Lactate	2.3 (1.4–3.9)	2.3 (1.4–4.0)	0.779

*CCU* coronary care unit, *CHF* chronic heart failure, *CKD* chronic kidney disease, *SICU* surgery intensive care unit, *SOFA* sequential organ failure assessment, *WBC* white blood cell.

Outliers were handled using a capping method, with values above the 99th percentile (P99) replaced by P99 and values below the 1st percentile (P1) replaced by P1 (Fig. S1). Missing data for all baseline variables did not exceed 30% (Fig. S2), and multiple imputation was performed using the MICE package^15^. For numerical variables, the worst values within the first 96 h of ICU admission were used. For variables with both upper and lower bounds (e.g., serum sodium, serum potassium), we selected the value furthest from the normal range using a custom SQL aggregation function (“Farthest”; see supplementary materials). For variables with only an upper bound (e.g., SOFA score, lactate, liver enzymes), the maximum value was chosen, while the minimum value was used for variables with only a lower bound (e.g., PaO_2_, PaO_2_/FiO_2_).

### Statistical analysis

We compared and described baseline characteristics across patient groups. Continuous variables were reported as mean (standard deviation) or median (interquartile range), with differences assessed using the t-test or Mann–Whitney U test, respectively. Categorical variables were presented as counts (percentages), and group comparisons were made using the chi-square test.

In our study, Latent Class Mixed Models (LCMM) were employed to analyze longitudinal time-series data for the identification of distinct latent subgroups^16^. We applied LCMM to classify patients in the training cohort based on their leukocyte count trajectories during the first 96 h of ICU admission. Models with 2–10 classes were tested, and the optimal model was selected using the Akaike Information Criteria(AIC) and the Bayesian Information Criterion (BIC) minimization. To test whether the selected categorical model was the best choice for this study, we conducted a multi-dimensional evaluation. Each patient was assigned to the subgroup with the highest probability, and posterior probabilities were used to evaluate the accuracy of these assignments. We also employed Vuong’s Likelihood Ratio Test (VLMR) to assess the goodness-of-fit between different categorical models. Additionally, we calculated the Mean Entropy and Normalized Entropy to evaluate the model’s classification stability and determinacy.

The same model selection method was applied to the validation cohort, and the model was subjected to the same multi-dimensional evaluation after subtype classification. The best classification model was consistently obtained in both the development and validation cohorts. To assess the impact of different leukocyte trajectories on 28-day mortality, Cox regression models and Kaplan–Meier survival curves were constructed. Additionally, logistic regression was performed to examine the association between subgroups and the use of vasopressors, mechanical ventilation, CRRT within 7 days, and 28-day mortality. To ensure the independent effect of subgroup classification on outcomes, multivariate regression analyses were conducted, adjusting for all baseline variables. We further developed an XGBoost model to early predict the high-risk mortality subtypes on patients’ICU admission. The candidate predictive factors included demographics, comorbidities, SOFA score, laboratory indicators, and vital signs. After confirming that the model had good predictive ability, the Shapley additive explanations (SHAP) was used to assess the predictive contribution of variables for high-mortality subtypes.

All statistical analyses were performed using R software (version 4.3.3), and LCMM models were constructed with the “lcmm” package^17^.

## Results

In the development cohort, 7410 patients were included to build the classification model. Based on Akaike Information Criteria(AIC) and Bayesian Information Criterion (BIC) comparisons, the model with eight groups provided the best fit (Table S1). The results of the multi-dimensional evaluation conducted for the model are as follows (Fig. S5, Tables S2 and S3). The posterior probabilities for this model ranged from 0.71 to 0.90, exceeding the acceptance threshold of 0.7, indicating an acceptable model fit. VLMR test for 8 versus 7 classes has the results of “*p* < 0.001”. However, the result of VLMR test between the 8-class and 9-class models was “*p*  = 0.11”. The 8-class model exhibited a lower Mean Entropy (0.483) and a higher Normalized Entropy (0.768). These performance metrics identified the 8-class model as the optimal classification model.

For external validation, 7564 patients were analyzed to build the classification model. The eight distinct leukocyte trajectory groups also exhibited the lowest AIC and BIC values(Table S1). Similarly, the model was subjected to multi-dimensional evaluation (Fig. S5, Tables S2 and S3). Posterior probabilities ranged from 0.72 to 0.90. VLMR test for 8 versus 7 classes has the results of “*p* < 0.001”. However, the result of VLMR test between the 8-class and 9-class models was “*p* = 0.09”. The 8-class model exhibited a lower Mean Entropy (0.524) and a higher Normalized Entropy (0.748). These metrics further validated the 8-class model as the optimal classification model. Further statistical analysis revealed significant differences in clinical characteristics and outcomes across the eight subtypes, both in the development and validation cohorts, supporting the model’s external validity.

**Fig. 2:**
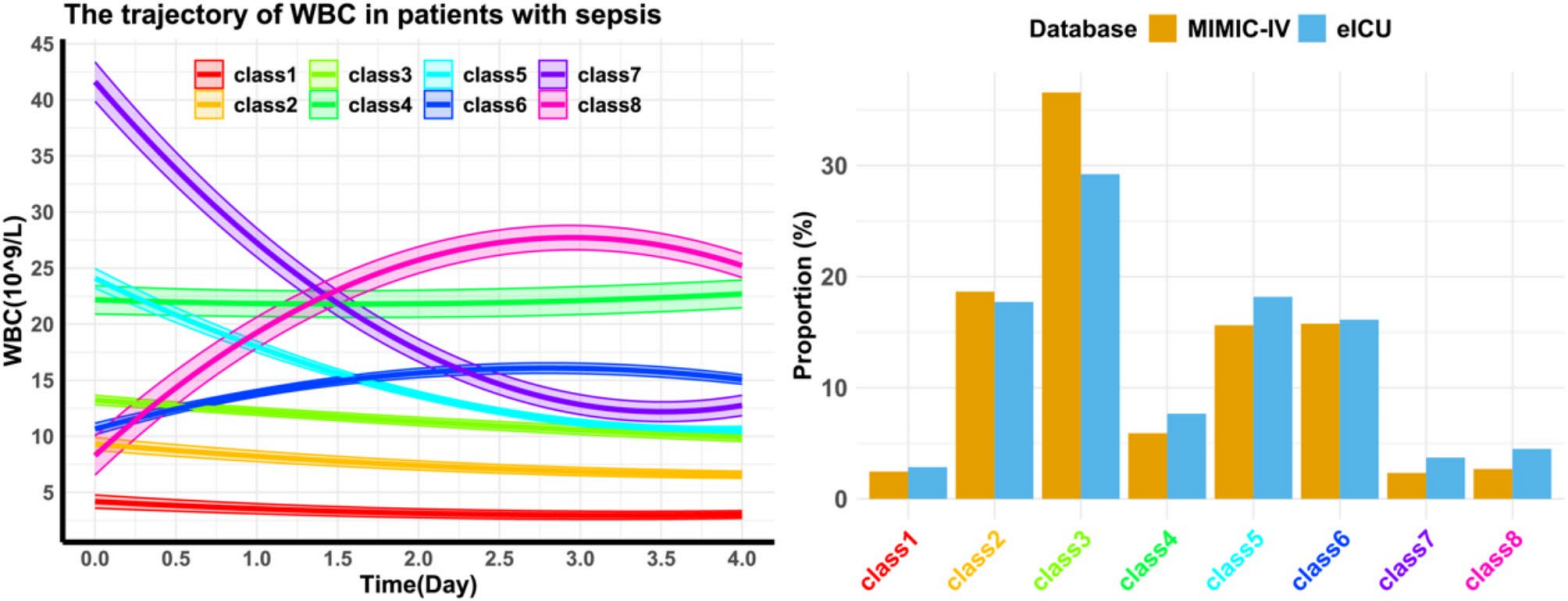
WBC Trajectory plot (left) and the proportion of participants(right). Abbreviation: *WBC* white blood cell.

As shown in Fig. 2, the early leukocyte trajectories after ICU admission and the proportion of patients in each group in both cohorts were as follows:

Class 1 (red, stable, low, 2.5%/2.9%): Consistently low leukocyte levels.

Class 2 (yellow, stable, normal, 18.7%/17.7%): Stable leukocyte levels within the normal range.

Class 3 (yellow-green, stable, high, 36.6%/29.2%): Slightly elevated leukocyte levels with minimal fluctuations.

Class 4 (green, stable, very high, 5.9%/7.7%): Persistently high leukocyte levels.

Class 5 (light blue, decreasing, high, 5.6%/18.2%): Elevated leukocyte levels with a decreasing trend.

Class 6 (dark blue, increasing, high, 15.8%/16.1%): Slightly elevated leukocyte levels with an increasing trend.

Class 7 (purple, decreasing, very high, 2.3%/3.7%): Extremely high leukocyte levels that rapidly decreased.

Class 8 (magenta, increasing, very high, 2.7%/4.5%): Initially normal leukocyte levels that sharply increased.

Class 3 was the most prevalent (36.6%/29.2%), followed by Class 2 (18.7%/17.7%), Class 5 (15.6%/18.2%), and Class 6 (15.8%/16.1%), which were nearly equal in proportion. These results suggest that most sepsis patients have elevated leukocyte levels, with varying degrees of fluctuation.

**Fig. 3:**
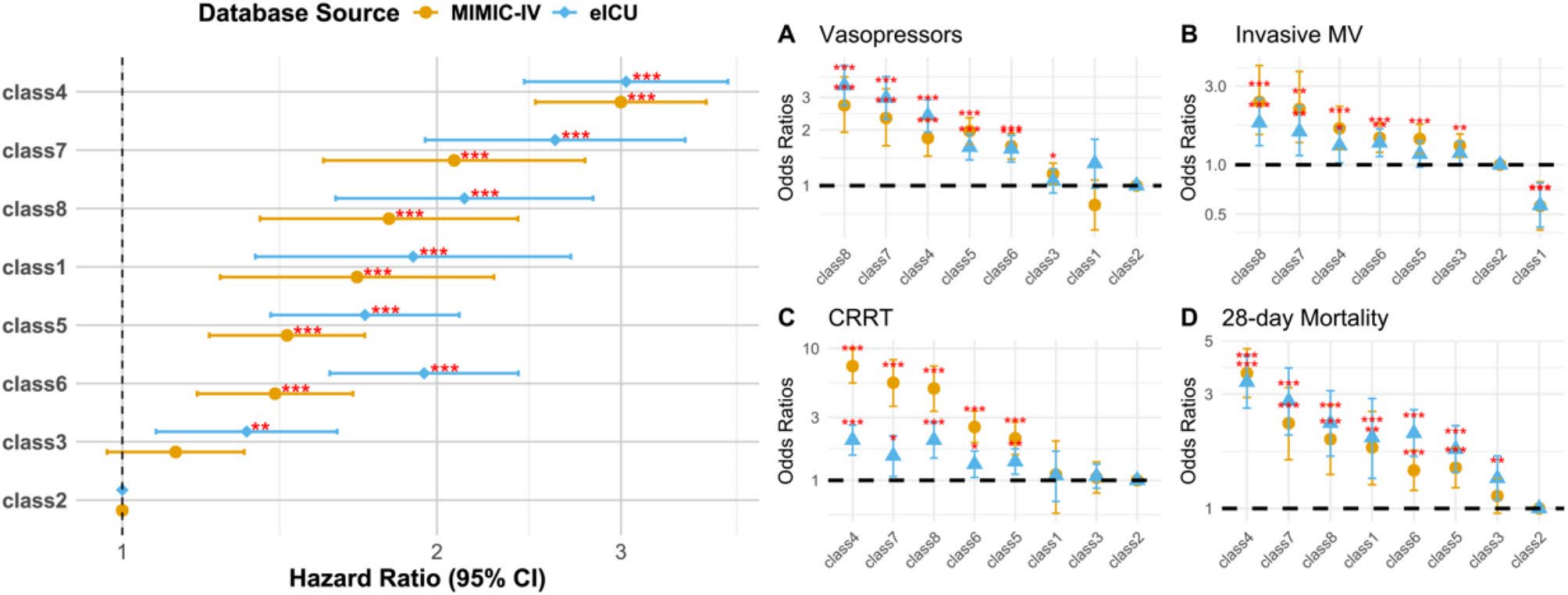
Forest Plot presentation: univariable cox regression and univariable logistic regression results. Abbreviation: *HR* Hazards ratio, *OR* odds ratios.

**Fig. 4:**
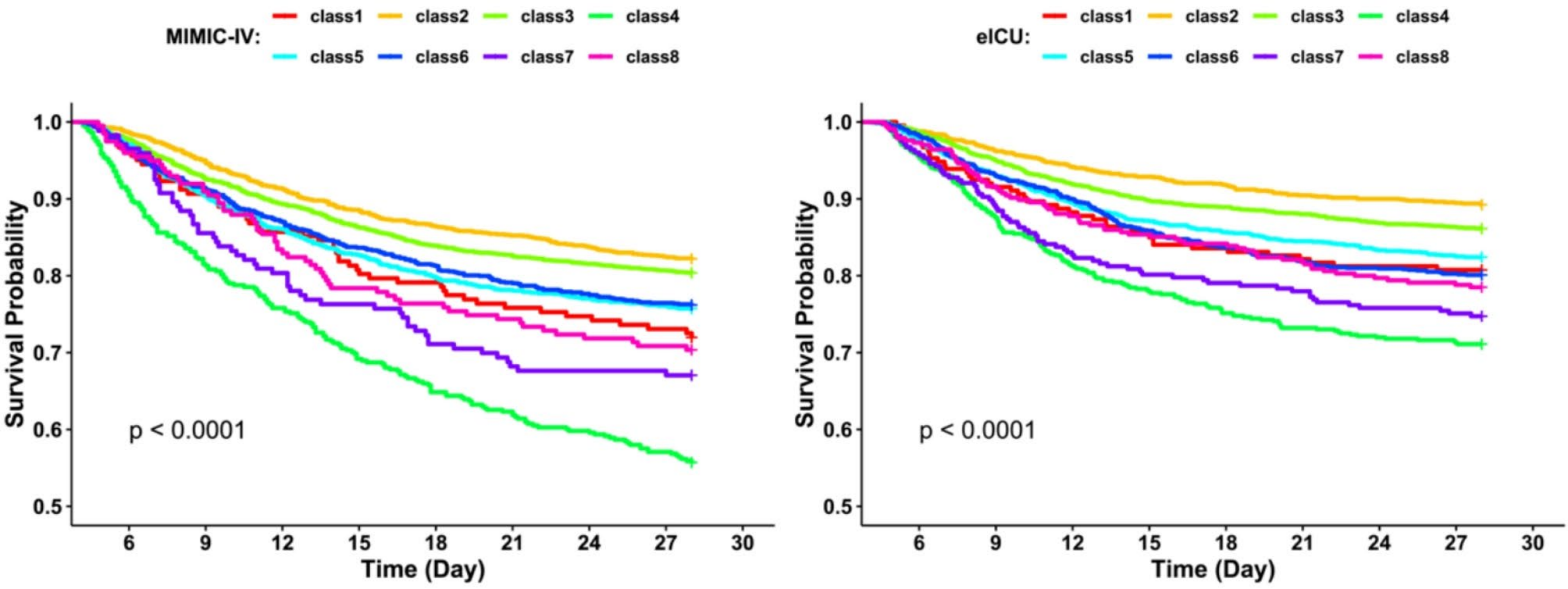
Kaplan–Meier curves of eight different dynamic WBC trajectory patterns.

**Fig. 5:**
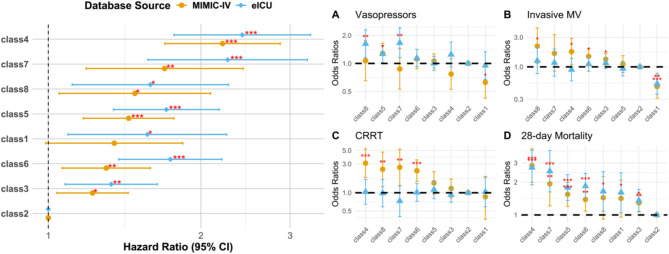
Forest Plot presentation: multivariable cox regression and multivariable logistic regression adjusted for all variables. Abbreviation: *HR* Hazards ratio, *OR* odds ratios.

Relationship Between Classifications and Outcomes:

In the development cohort, 1698 patients died within 28 days, while the validation cohort had 1,269 deaths. A Cox regression model (Fig. 3 and Table S6) and survival curves (Fig. 4) were generated, with Class 2 (the group with stable, normal leukocyte levels) serving as the reference group due to its lowest mortality risk. In contrast, Class 4 (persistently very high leukocyte levels) had the highest mortality risk (HR 3.00; 95% CI 2.48–3.62; *p* < 0.001), followed by Class 7 (rapidly decreasing, very high leukocyte levels, HR 2.08; 95% CI 1.56–2.77; *p* < 0.001), Class 8 (sharply increasing, very high leukocyte levels, HR 1.80; 95% CI 1.35–2.39; *p* < 0.001), and Class 1 (consistently low leukocyte levels, HR 1.68; 95% CI 1.24–2.27; *p* < 0.001). These associations remained significant even after adjusting for baseline variables, including static leukocyte measurements (Fig. 5/Table S8).

The logistic regression model further explored the relationship between leukocyte subtypes and the need for life-support therapies. Subtypes with higher leukocyte levels, particularly Class 4, Class 7, and Class 8, were associated with increased use of life-support treatments, mirroring the trends observed in the Cox model. However, the relationship between subtype classification and life-support use was less pronounced in the multivariate regression model, suggesting that this association may not be fully independent (Fig. 5/Table S9).

Interestingly, despite Class 1 having a higher mortality risk in the Cox model, its use of life-support therapies was not significantly different from Class 5 (the reference group). Class 1 was even associated with a lower need for invasive mechanical ventilation (development cohort: OR 0.56; 95% CI 0.40–0.79; *p* < 0.001; validation cohort: OR 0.57; 95% CI 0.42–0.77; *p* < 0.001), a finding that persisted after multivariate adjustment (Fig. 5).

When examining leukocyte trajectory trends, the impact on outcomes was not entirely dependent on the trajectory pattern. For instance, Class 5 (decreasing, high leukocyte levels) and Class 6 (increasing, high leukocyte levels), which had similar overall leukocyte levels within the first 4 days, showed comparable 28-day mortality risks (development cohort: HR 1.44; 95% CI 1.21–1.71 versus HR 1.40; 95% CI 1.18–1.66; validation cohort: HR 1.71; 95% CI 1.39–2.10 versus HR 1.94; 95% CI 1.58–2.39). A similar trend was observed between Class 7 and Class 8 (development cohort: HR 2.08; 95% CI 1.56–2.77 versus HR 1.80; 95% CI 1.35–2.39; validation cohort: HR 2.60; 95% CI 1.95–3.46 versus HR 2.12; 95% CI 1.60–2.82).

Subtypes Reproducibility And Prediction:

We further trained an XGBoost model to predict subtypes based on patient characteristics upon ICU admission. To evaluate the predictive performance of the model, we calculated the AUC values for the four high-mortality classifications (Class 1, Class 4, Class 7, and Class 8) in both the development cohort (Dev-Cohort) and validation cohort (Val-Cohort) (Fig. S6). The results showed that all AUC values exceeded 0.8, with similar AUCs between the validation cohort (purple curve) and the development cohort (green curve). Other performance metrics (Table S15) indicated that for Class 1 and 4, PPV was greater than 0.7, while for Class 7 and 8, PPV was close to 0.7. However, considering other performance metrics, NPV exceeded 0.8, accuracy exceeded 0.8, and balanced accuracy was above 0.75 across all classifications in both cohorts, demonstrating strong predictive performance of the model.

**Fig. 6:**
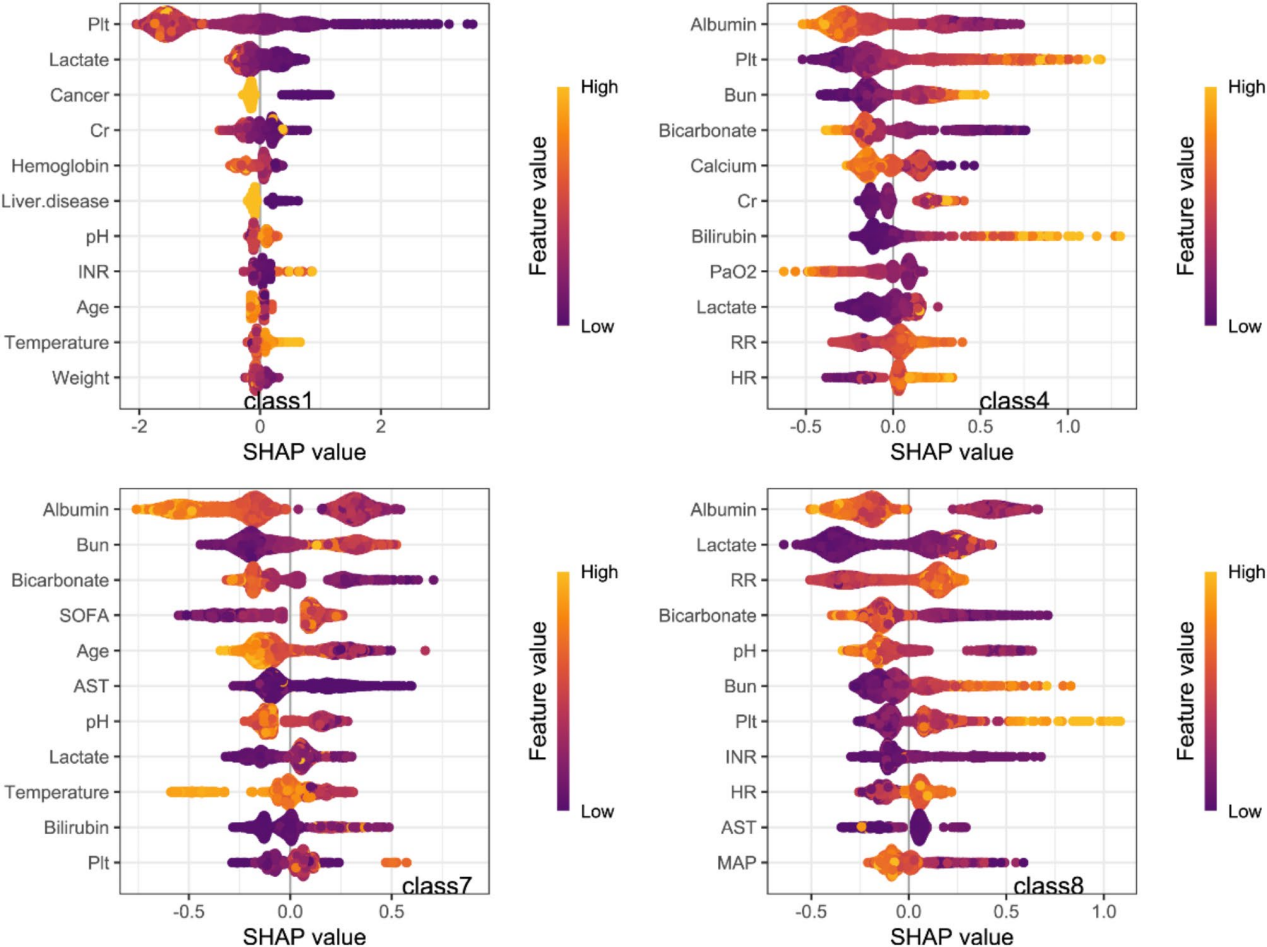
Shap value visualization: features of four subtypes based on model predictions. Abbreviation: *AST* aspartate aminotransferase, *BUN* blood urea nitrogen, *CR* creatinine, *HR* heart rate, *INR* international normalized ratio, *MAP* mean arterial pressure, *PLT* platelet, *pH* potential of hydrogen, *RR* respiratory rate.

When using the trained XGBoost model to predict high-risk mortality subtypes, SHAP values exhibite that each has distinct clinical features (Fig. 6). Variables such as lactate, bicarbonate, platelet count, albumin, and PaO2 have a significant impact on predicting subtypes related to clinical outcomes. Group 1 (Consistently low leukocyte levels) was characterized by lower hemoglobin, platelet count, and creatinine, as well as a lower prevalence of cancer and liver disease. In contrast, low albumin, high platelet count, high creatinine, and high bilirubin made a significant contribution to predicting Group 4 (Persistently high leukocyte levels). Low albumin, higher blood urea nitrogen (BUN), high SOFA score, and younger age were stronger predictors of Group 7 (Extremely high leukocyte levels that rapidly decreased), while low albumin, low lactate, high heart rate, low pH, high BUN, and high platelet count were more characteristic of Group 8 (Initially normal leukocyte levels that sharply increased).

## Discussion

In this study, we applied a machine learning approach, Latent Class Mixed Models (LCMM), to analyze dynamic time-series data and identify potential leukocyte trajectory subtypes in sepsis patients. In the results, we observed that the 8-class model was selected as the optimal classification model in the development cohort based on the minimum AIC and BIC values (Table S1). A multidimensional evaluation of the classification performance of this optimal model (Fig. S5, Tables S2, S3) indicated that the 8-class model was the best choice: in both cohorts, the posterior probability exceeded the acceptable threshold of 0.7, demonstrating the robustness of the model.

Furthermore, VLMR test confirmed that the 8-class model had a significantly better fit than the 7-class model (*p* < 0.001). However, there was no significant difference between the 8-class and 9-class models, suggesting that the 8-class model was sufficiently effective without the risk of overfitting due to an excessive number of classes. The 8-class model also exhibited a lower Mean Entropy and higher Normalized Entropy, indicating higher classification stability and lower uncertainty.Further data analysis showed that the regression analyses in the validation cohort aligned with the results from the training cohort. Notably, patients with persistently elevated leukocyte levels had the poorest clinical outcomes, a finding that remained consistent even after adjusting for baseline variables, including static leukocyte measurements. Additionally, we developed a multivariable prediction model to identify high-risk mortality subtypes at ICU admission. The predictive performance metrics (Table S15) indicated that the model effectively predicted the baseline characteristics of high-mortality subphenotypes. SHAP values further demonstrated that the impact of different combinations of feature variables on high-mortality subphenotype classification was highly stable (Fig. 6).

In critical care medicine, syndromes are commonly used to categorize patient groups in both clinical practice and research. However, as our understanding of disease complexity deepens, there is a growing recognition of the need for precision medicine. Sepsis, a highly heterogeneous condition, exemplifies this challenge, as identifying distinct clinical subtypes is essential for tailoring treatment and improving prognostic assessments^10^. For instance, Bhavani et al. identified four sepsis subtypes based on vital sign trajectories, revealing differences in prognosis and fluid therapy response^8^. Similarly, sepsis subtypes have been defined using organ failure trajectories derived from SOFA scores. Van Amstel et al. explored the relationships between different sepsis classification methods, finding little overlap, except for some similarities between Mars2 and SRS1 in terms of host response biomarkers (*p* = 0.079–0.424)^5^.

A similar study previously examined leukocyte trajectories in septic shock patients, analyzing 917 cases and identifying seven distinct subgroups. Consistent with our findings, the subgroup with the highest mortality in that study (subgroup five) closely resembled our Class 4 trajectory, which was strongly associated with poor outcomes^18^. However, a notable difference in our study is the identification of a low leukocyte subgroup (Class 1), which we interpret as an immunosuppressive phenotype. This subgroup had unique baseline characteristics, including lower values of platelet count, hemoglobin, lactate and creatinine, as well as a relatively lower prevalence of cancer and liver disease.(see Table S10). The SHAP values indicate that low values of these feature variables have a stable impact on the classification of this subphenotype (Fig. 6). Patients in this group who received invasive mechanical ventilation were significantly fewer than in other groups (see Table S9). The Class 1 subgroup, characterized by low leukocyte trajectory, relatively stable metabolism, and normal renal function, suggests that these patients lack typical acute immune responses and are less likely to develop respiratory failure symptoms. Alternatively, these patients may have opted for a more conservative approach, avoiding invasive interventions like intubation^19–22^.

It’s important to note that immunosuppression in these patients may not be entirely attributable to their comorbidities but could also be a consequence of sepsis itself, highlighting the need for close attention to this phenotype in clinical practice^23,24^.

We compared Class 5 and Class 6, as well as Class 7 and Class 8, which had similar areas under the trajectory curve (indicating comparable average leukocyte levels), but showed opposite trends following ICU admission. Despite these contrasting trends, there was no statistically significant difference in direct mortality risk between these groups, and logistic regression analysis confirmed similar findings. This lack of difference may reflect the timing of infection onset: Classes 5 and 7, which displayed a decreasing trend in leukocyte levels, likely had infections prior to ICU admission. In these cases, inflammation may have been better controlled after ICU admission, resulting in a marked reduction in leukocyte levels.

Class 4, representing 5.9% of the development cohort and 7.7% of the validation cohort, exhibited the highest mortality risk. This aligns with clinical observations that persistently elevated leukocyte levels are often associated with severe, hard-to-control infections^4^, Interestingly, despite Classes 7 and 8 displaying higher peak leukocyte levels than Class 4, their outcomes were relatively better. This trend echoes findings from a study by Xu Wang et al., which examined procalcitonin trajectories in sepsis patients during the first 7 days of ICU admission. They found that patients with persistently low procalcitonin levels had worse outcomes compared to those with higher levels but a decreasing trend—paralleling the results in our study^25^.

Using the XGBoost algorithm combined with SHAP method, we captured the clinical characteristics of high-mortality risk subgroups (Class 1, 4, 7, and 8) at ICU admission. By plotting ROC curves and calculating AUC values, we evaluated the model’s performance. Overall, the model demonstrated high AUCs in the development set (> 0.82) and maintained good generalization ability in the validation set (AUC range: 0.818–0.878), indicating strong discriminative power across different white blood cell trajectory subtypes, excellent predictive performance, and consistent external performance—reflecting good robustness and external validity.

SHAP interpretation method revealed that lactate and albumin were the most influential variables in determining white blood cell trajectory subtypes. Patients with lower albumin and higher lactate levels were more likely to belong to high-risk subgroups. Platelet count (Plt), blood urea nitrogen (BUN), and bicarbonate also showed substantial contributions across several classes, suggesting that coagulation status, renal function, and acid-base balance play important roles in sepsis subtype classification. Additionally, respiratory rate (RR) and PaO_2_ contributed notably in specific subtypes, highlighting oxygenation status as a key feature in certain groups^26^.

Class 1 (persistently low pattern) exhibits distinct clinical characteristics and represents a hypoinflammatory subtype of sepsis, suggesting a potential state of immunosuppression. From a theoretical perspective, immunostimulatory therapies may be beneficial for this group of patients. Common strategies include immunostimulatory cytokines and growth factors (such as GM-CSF, G-CSF, and IL-7), intravenous immunoglobulin (IVIG), mesenchymal stem cells (MSCs), and immune checkpoint inhibitors (e.g., PD-1 inhibitors)^27^.

The remaining three high-mortality risk subgroups exhibited overall white blood cell levels significantly above the normal range and shared similar clinical characteristics. At ICU admission, hypoalbuminemia emerged as their most prominent feature, accompanied by elevated lactate levels and reduced bicarbonate concentrations (base levels), indicating early hyperlactatemia, poor tissue perfusion, and metabolic acidosis.

Studies have shown that hypoalbuminemia in sepsis is associated with increased albumin clearance, and early albumin resuscitation may improve outcomes^28,29^. For these patients, timely albumin supplementation and fluid resuscitation may have unique therapeutic benefits.Additionally, Class 4 and Class 8 were characterized by increased respiratory rate and tachycardia, suggesting a stronger early stress response upon ICU admission in these subgroups.

This study has several limitations. First, it was difficult to assess differences in treatment responses among the identified subtypes, which limits our understanding of how these classifications may inform therapeutic strategies. Future prospective studies are needed to validate the clinical utility of these classifications. Second, to ensure model fit and accuracy, we included only patients who stayed in the ICU for more than 4 days and were diagnosed with sepsis within 24 h of admission. The effectiveness of this classification for excluded patients, such as those with shorter ICU stays or later sepsis diagnoses, remains unclear. Third, we did not collect data on other inflammatory markers, such as C-reactive protein (CRP), procalcitonin (PCT), or heparin-binding protein (HBP), which limits a more comprehensive evaluation of the inflammatory status in these patients. Finally, the retrospective nature of this study may limit the applicability of these findings in prospective clinical settings.

## Conclusion

Using the Latent Class Mixed Model (LCMM), we identified eight distinct sepsis subtypes based on leukocyte trajectories within the first 96 h of ICU admission. These subtypes exhibited significant differences in clinical outcomes and organ support requirements, proving to be independent prognostic indicators for sepsis, beyond static leukocyte measurements. External validation with an independent cohort confirmed the robustness of these findings. The XGBoost prediction model constructed using baseline characteristics upon ICU admission is able to predict high-mortality phenotypes based on baseline variables. The hyperinflammatory and hypoinflammatory subtypes exhibit distinct clinical characteristics in the early phase of ICU admission. Further research is needed to explore the clinical relevance of these subtypes, particularly their potential overlap and interaction with existing sepsis classifications, to enhance personalized treatment strategies.

## Electronic supplementary material

Below is the link to the electronic supplementary material.

Supplementary Material 1

Supplementary Material 2

Supplementary Material 3

## Data Availability

Our data was obtained from MIMIC-IV2.2 and eICU-CRD databases，which is available in PhysioNet (https://physionet.org), thus no more permission was required.

## Abbreviations

AIC: Akaike information criteria
BIC: Bayesian information criterion
CCU: Coronary care unit
CHF: Chronic heart failure
CI: confidence interval
CKD: Chronic kidney disease
CRP: C-reactive protein
CRRT: Continuous renal replacement therapy
HBP: Heparin-binding protein
HR: Hazards ratio
ICU: Intensive care unit
LCMM: Latent class mixed models
MAP: Mean arterial pressure
MIMIC: The medical information mart for intensive care database
MV: Mechanical ventilation
PCT: Procalcitonin
SD: Standard deviations
SHAP: Shapley additive explanations
SICU: Surgery intensive care unit
SOFA: Sequential organ failure assessment
SQL: Structured query language
VLMR: Vuong’s likelihood ratio test
WBC: White blood cell
