# Supplementary material for: Clinical subtypes identification and feature recognition of sepsis leukocyte trajectories based on machine learning
Source: Sci Rep. 2025 Apr 10;15:12291. doi: 10.1038/s41598-025-96718-9 (PMC11986166; doi:10.1038/s41598-025-96718-9)
Supplement: Supplementary file 1 — Supplementary Material 1 [file 41598_2025_96718_MOESM1_ESM.docx]

**Clinical Subtypes Identification and Feature Recognition of Sepsis Leukocyte Trajectories Based on Machine Learning**

^a†^ShengHui Miao, ^b†^YiJing Liu, ^a^Min Li,^c*^Jing Yan

†. ShengHui Miao and YiJing Liu contributed equally to this work.

a. The Fourth Affiliated Hospital, International Institutes of Medicine, Zhejiang University School of Medicine, YiWu 322000, China

b. Department of Second Clinical Medical College, Zhejiang Chinese Medicine University, Hangzhou 310053, Zhejiang, China.

c. Zhejiang Hospital, Zhejiang University School of Medicine, Lingyin Road 12, Hangzhou 310013, Zhejiang, China.

Corresponding author

^c*^Jing Yan

Zhejiang Hospital, Zhejiang University School of Medicine, Lingyin Road 12, Hangzhou 310013, Zhejiang, China. Tel: +86-571-81595216

E-mail: [yanjing201801@163.com](mailto:yanjing201801@163.com)


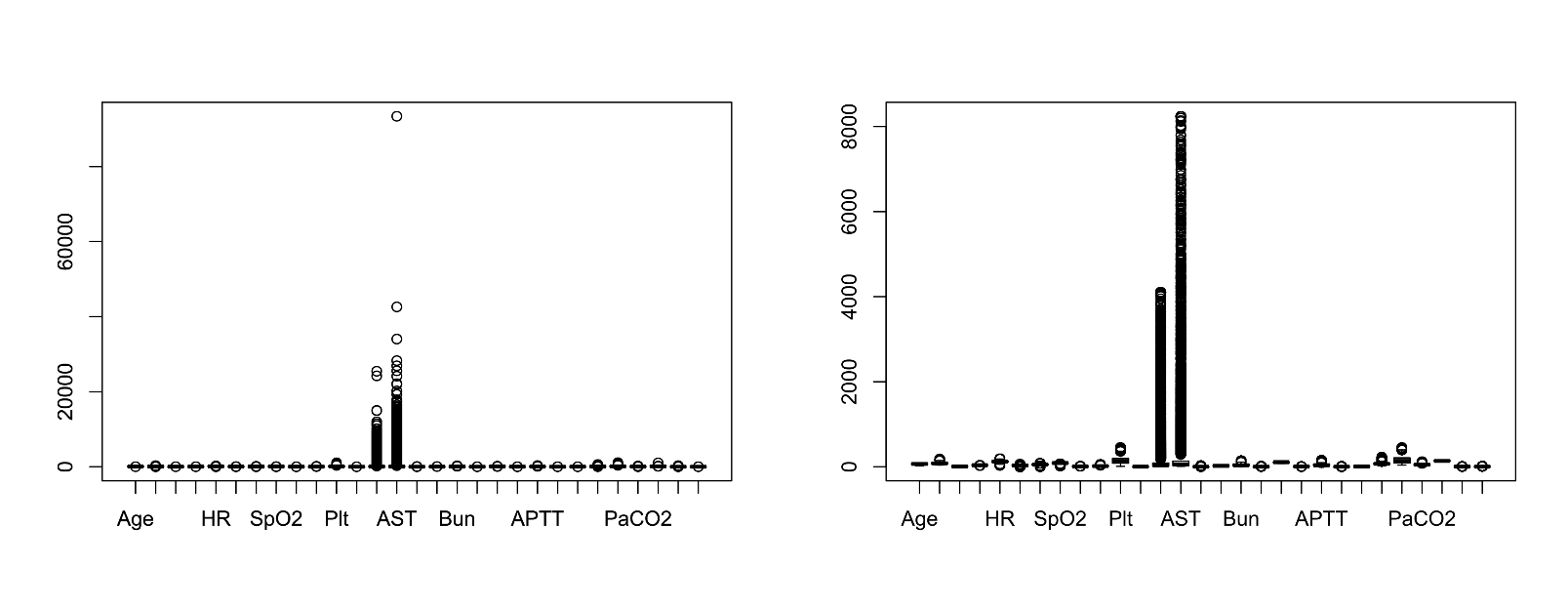


**Figure S1: Analysis of outliers in numerical variables(left) and results after processing(right).**


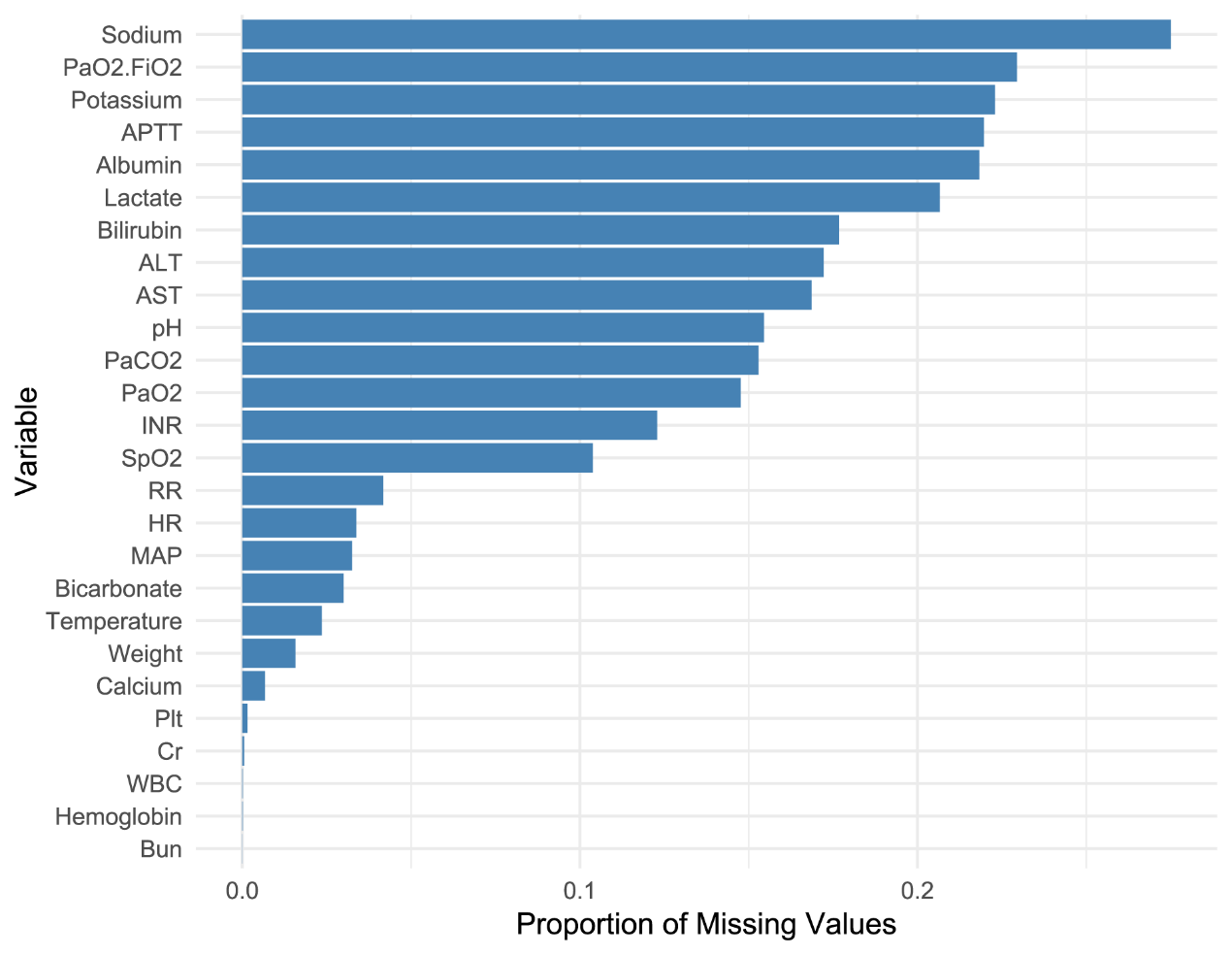


**Figure S2: The proportion of missing values in variable.**


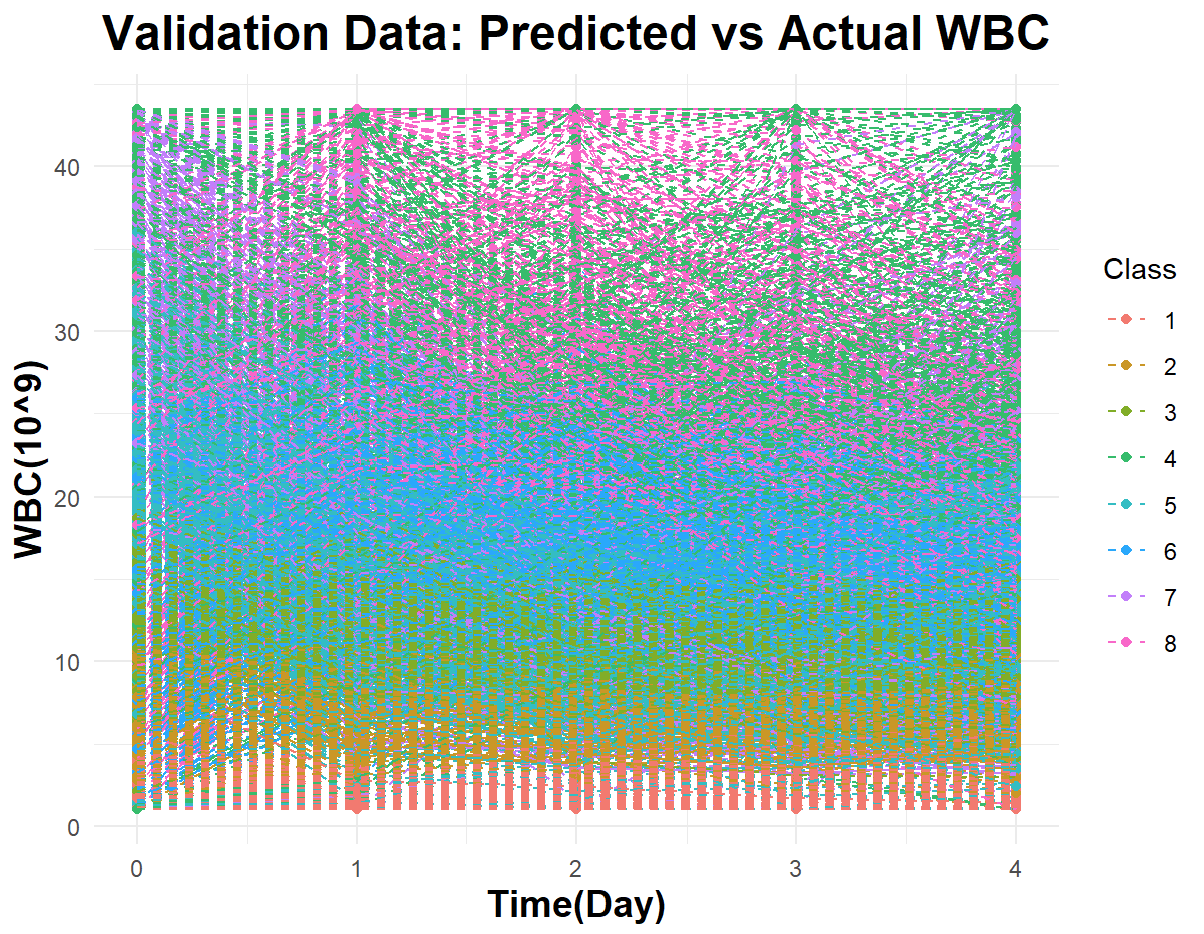


**Figure S3: Predicted vs Actual WBC trajectories in the first four days in Validation Data.**

**Figure S5: Forest Plot Presentation: Multivariable Cox Regression and Multivariable Logistic Regression Adjusted for Demographic Variables.**


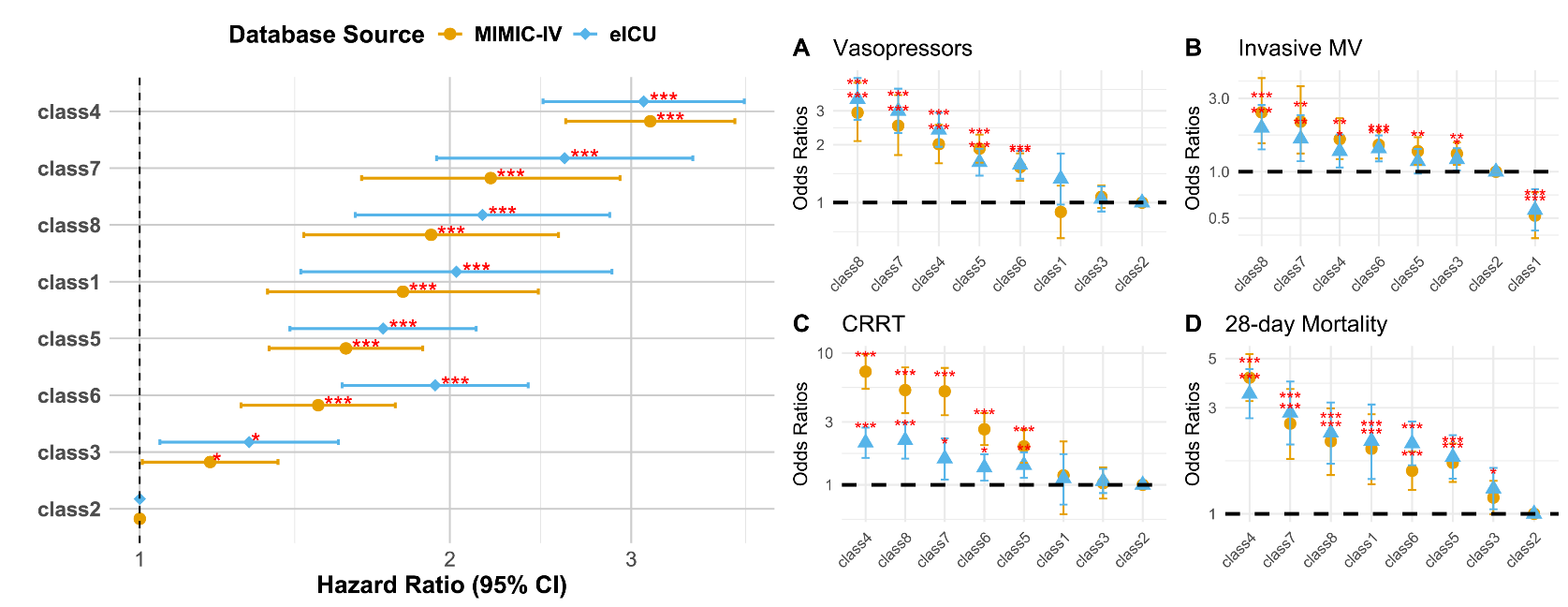


**Figure S4: Forest Plot Presentation: Multivariable Cox Regression and Multivariable Logistic Regression Adjusted for Demographic Variables.**


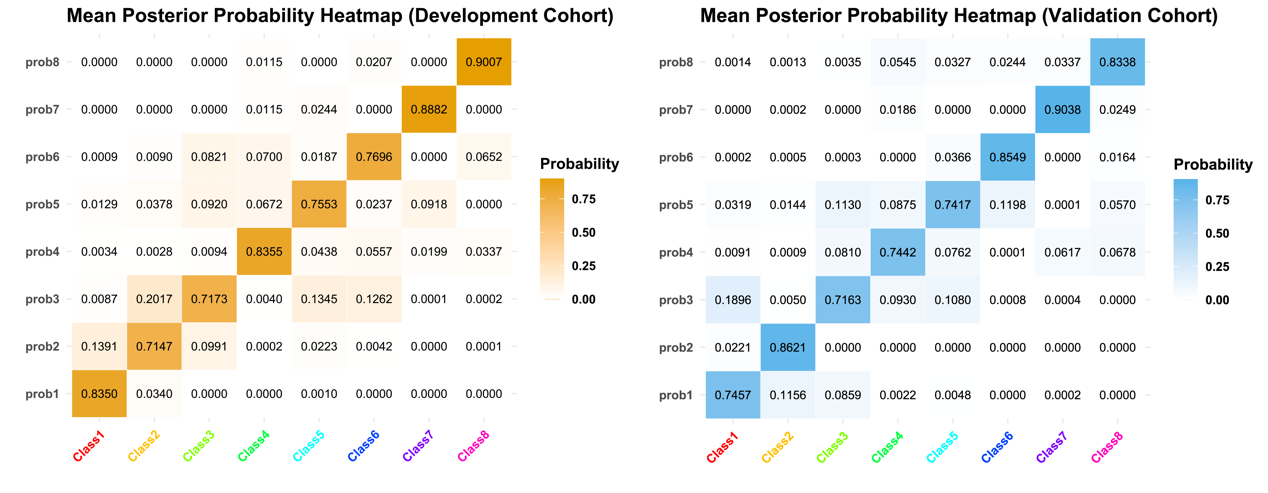


**Figure S5: Mean posterior probability heatmap of the eight WBC trajectory patterns in Development Cohort and Validation Cohort.**


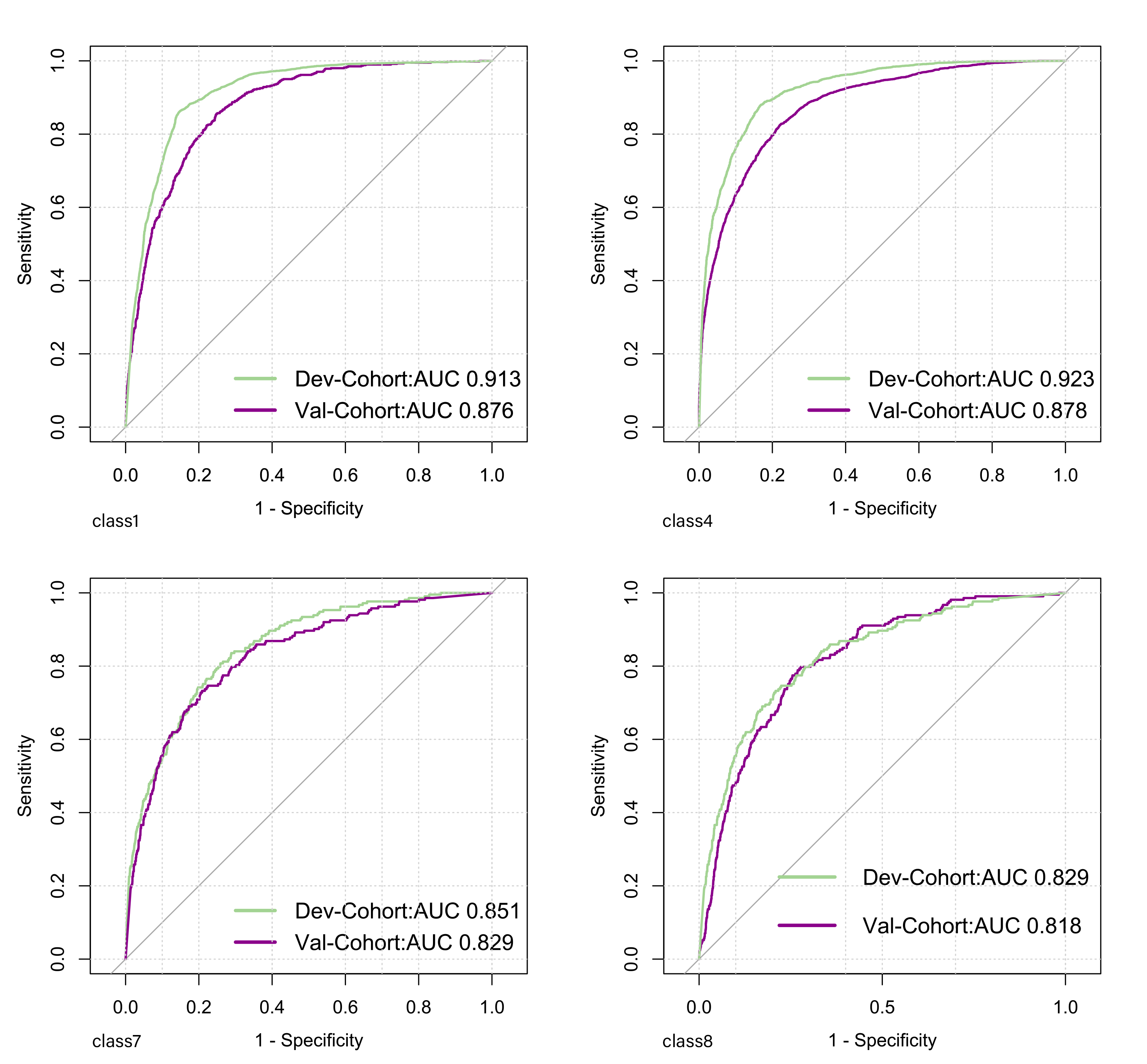


**Figure S6: AUC Values for Four Subtypes with the Highest Mortality Rates.**
