# Supplementary material for: Clinical subtypes identification and feature recognition of sepsis leukocyte trajectories based on machine learning
Source: Sci Rep. 2025 Apr 10;15:12291. doi: 10.1038/s41598-025-96718-9 (PMC11986166; doi:10.1038/s41598-025-96718-9)
Supplement: Supplementary file 2 — Supplementary Material 2 [file 41598_2025_96718_MOESM2_ESM.docx]

**Clinical Subtypes Identification and Feature Recognition of Sepsis Leukocyte Trajectories Based on Machine Learning**

^a†^ShengHui Miao, ^b†^YiJing Liu, ^a^Min Li,^c*^Jing Yan

†. ShengHui Miao and YiJing Liu contributed equally to this work.

a. The Fourth Affiliated Hospital, International Institutes of Medicine, Zhejiang University School of Medicine, YiWu 322000, China

b. Department of Second Clinical Medical College, Zhejiang Chinese Medicine University, Hangzhou 310053, Zhejiang, China.

c. Zhejiang Hospital, Zhejiang University School of Medicine, Lingyin Road 12, Hangzhou 310013, Zhejiang, China.

Corresponding author

^c*^Jing Yan

Zhejiang Hospital, Zhejiang University School of Medicine, Lingyin Road 12, Hangzhou 310013, Zhejiang, China. Tel: +86-571-81595216

E-mail: [yanjing201801@163.com](mailto:yanjing201801@163.com)

**Table S1: Fit statistics for latent class mixed models in Development Cohort and Validation Cohort.**

|  | **Development Cohort:** | | **Validation Cohort:** | |
| --- | --- | --- | --- | --- |
| Number Of classes | AIC | BIC | AIC | BIC |
| 2 | 404298.9 | 404390.3 | 375239.5 | 375329.3 |
| 3 | 396422.7 | 396552.1 | 368527.2 | 368654.4 |
| 4 | 395120.3 | 395287.8 | 367293.9 | 367458.6 |
| 5 | 394349.6 | 394555.2 | 366662.0 | 366864.0 |
| 6 | 393370.5 | 393614.1 | 365265.4 | 365504.9 |
| 7 | 391421.9 | 391703.5 | 364259.2 | 364536.2 |
| **8** | **390968.1** | **391287.8** | **363703.3** | **364017.6** |
| 9 | 391247.2 | 392496.1 | 364107.1 | 364588.1 |
| 10 | 392869.7 | 393838.3 | 365001.8 | 365728.2 |
| Abbreviation: AIC, Akaike information criteria；BIC, Bayesian information criteria； | | | | |

**Table S2: VLMR test for optimal class number in Development Cohort and Validation Cohort.**

|  | **Development Cohort:** | **Validation Cohort:** |
| --- | --- | --- |
| VLMR test for 8 vs 7 classes: | Statistic=463.7709(P-value < 2.22e-16) | Statistic = 397.2368(P-value < 2.22e-16) |
| VLMR test for 9 vs 8 classes | Statistic=2.302172(P-value = 0.11) | Statistic = 2.712378(P-value = 0.09) |

**Table S3: Entropy Metrics in Development Cohort and Validation Cohort.**

|  | **Development Cohort:** | **Validation Cohort:** |
| --- | --- | --- |
| Mean Entropy | 0.483 | 0.524 |
| Normalized Entropy | 0.768 | 0.748 |

**Table S4: Multivariable Cox Regression Analysis between WBC trajectory and 28day-mortality adjusted by Demographic Characteristics***

| **Database** | **CLASS** | **HR(95%CI; p value)** | **Significance** |
| --- | --- | --- | --- |
| MIMIC-IV | class2 | HR 1.00; 95% CI 1.00–1.00; p = NA |  |
| MIMIC-IV | class1 | HR 1.80; 95% CI 1.33–2.44; p = <0.001 | *** |
| MIMIC-IV | class3 | HR 1.17; 95% CI 1.01–1.36; p = 0.042 | * |
| MIMIC-IV | class4 | HR 3.13; 95% CI 2.59–3.78; p = <0.001 | *** |
| MIMIC-IV | class5 | HR 1.59; 95% CI 1.34–1.88; p = <0.001 | *** |
| MIMIC-IV | class6 | HR 1.49; 95% CI 1.25–1.77; p = <0.001 | *** |
| MIMIC-IV | class7 | HR 2.19; 95% CI 1.64–2.93; p = <0.001 | *** |
| MIMIC-IV | class8 | HR 1.92; 95% CI 1.44–2.55; p = <0.001 | *** |
| eICU | class2 | HR 1.00; 95% CI 1.00–1.00; p = NA |  |
| eICU | class1 | HR 2.03; 95% CI 1.43–2.87; p = <0.001 | *** |
| eICU | class3 | HR 1.28; 95% CI 1.05–1.56; p = 0.016 | * |
| eICU | class4 | HR 3.08; 95% CI 2.46–3.86; p = <0.001 | *** |
| eICU | class5 | HR 1.72; 95% CI 1.40–2.12; p = <0.001 | *** |
| eICU | class6 | HR 1.94; 95% CI 1.57–2.38; p = <0.001 | *** |
| eICU | class7 | HR 2.58; 95% CI 1.94–3.44; p = <0.001 | *** |
| eICU | class8 | HR 2.15; 95% CI 1.62–2.86; p = <0.001 | *** |

*including: age、sex、race、weight、icu-type.

Abbreviation: HR, Hazards ratio;

**Table S5: Multivariable Logistic Regression Analysis between WBC trajectory and secondary outcomes adjusted by Demographic Characteristics***

| Database | Variable | CLASS | OR (95% CI; p value) | Significance |
| --- | --- | --- | --- | --- |
| MIMIC-IV | Vasopressors | class2 | OR 1.00; 95% CI 1.00–1.00; p = NA | NA |
| MIMIC-IV | Vasopressors | class1 | OR 0.89; 95% CI 0.65–1.22; p = 0.487 |  |
| MIMIC-IV | Vasopressors | class3 | OR 1.07; 95% CI 0.94–1.23; p = 0.301 |  |
| MIMIC-IV | Vasopressors | class4 | OR 2.01; 95% CI 1.60–2.54; p = <0.001 | *** |
| MIMIC-IV | Vasopressors | class5 | OR 1.91; 95% CI 1.61–2.26; p = <0.001 | *** |
| MIMIC-IV | Vasopressors | class6 | OR 1.53; 95% CI 1.30–1.81; p = <0.001 | *** |
| MIMIC-IV | Vasopressors | class7 | OR 2.51; 95% CI 1.77–3.62; p = <0.001 | *** |
| MIMIC-IV | Vasopressors | class8 | OR 2.94; 95% CI 2.09–4.20; p = <0.001 | *** |
| eICU | Vasopressors | class2 | OR 1.00; 95% CI 1.00–1.00; p = NA | NA |
| eICU | Vasopressors | class1 | OR 1.33; 95% CI 0.98–1.80; p = 0.067 |  |
| eICU | Vasopressors | class3 | OR 1.05; 95% CI 0.90–1.21; p = 0.561 |  |
| eICU | Vasopressors | class4 | OR 2.39; 95% CI 1.95–2.93; p = <0.001 | *** |
| eICU | Vasopressors | class5 | OR 1.62; 95% CI 1.38–1.91; p = <0.001 | *** |
| eICU | Vasopressors | class6 | OR 1.57; 95% CI 1.33–1.86; p = <0.001 | *** |
| eICU | Vasopressors | class7 | OR 3.00; 95% CI 2.30–3.91; p = <0.001 | *** |
| eICU | Vasopressors | class8 | OR 3.45; 95% CI 2.69–4.44; p = <0.001 | *** |
| MIMIC-IV | Invasive.MV | class2 | OR 1.00; 95% CI 1.00–1.00; p = NA | NA |
| MIMIC-IV | Invasive.MV | class1 | OR 0.52; 95% CI 0.37–0.73; p = <0.001 | *** |
| MIMIC-IV | Invasive.MV | class3 | OR 1.31; 95% CI 1.11–1.55; p = 0.001 | ** |
| MIMIC-IV | Invasive.MV | class4 | OR 1.63; 95% CI 1.21–2.23; p = 0.002 | ** |
| MIMIC-IV | Invasive.MV | class5 | OR 1.36; 95% CI 1.11–1.68; p = 0.004 | ** |
| MIMIC-IV | Invasive.MV | class6 | OR 1.50; 95% CI 1.22–1.85; p = <0.001 | *** |
| MIMIC-IV | Invasive.MV | class7 | OR 2.12; 95% CI 1.31–3.59; p = 0.003 | ** |
| MIMIC-IV | Invasive.MV | class8 | OR 2.43; 95% CI 1.53–4.06; p = <0.001 | *** |
| eICU | Invasive.MV | class2 | OR 1.00; 95% CI 1.00–1.00; p = NA | NA |
| eICU | Invasive.MV | class1 | OR 0.56; 95% CI 0.41–0.77; p = <0.001 | *** |
| eICU | Invasive.MV | class3 | OR 1.20; 95% CI 1.02–1.42; p = 0.027 | * |
| eICU | Invasive.MV | class4 | OR 1.36; 95% CI 1.07–1.75; p = 0.014 | * |
| eICU | Invasive.MV | class5 | OR 1.17; 95% CI 0.97–1.41; p = 0.096 |  |
| eICU | Invasive.MV | class6 | OR 1.42; 95% CI 1.17–1.72; p = <0.001 | *** |
| eICU | Invasive.MV | class7 | OR 1.64; 95% CI 1.17–2.34; p = 0.005 | ** |
| eICU | Invasive.MV | class8 | OR 1.93; 95% CI 1.40–2.71; p = <0.001 | *** |
| MIMIC-IV | CRRT | class2 | OR 1.00; 95% CI 1.00–1.00; p = NA | NA |
| MIMIC-IV | CRRT | class1 | OR 1.18; 95% CI 0.60–2.13; p = 0.609 |  |
| MIMIC-IV | CRRT | class3 | OR 1.03; 95% CI 0.78–1.36; p = 0.842 |  |
| MIMIC-IV | CRRT | class4 | OR 7.23; 95% CI 5.34–9.84; p = <0.001 | *** |
| MIMIC-IV | CRRT | class5 | OR 1.96; 95% CI 1.47–2.62; p = <0.001 | *** |
| MIMIC-IV | CRRT | class6 | OR 2.64; 95% CI 2.00–3.51; p = <0.001 | *** |
| MIMIC-IV | CRRT | class7 | OR 5.13; 95% CI 3.37–7.73; p = <0.001 | *** |
| MIMIC-IV | CRRT | class8 | OR 5.23; 95% CI 3.50–7.78; p = <0.001 | *** |
| eICU | CRRT | class2 | OR 1.00; 95% CI 1.00–1.00; p = NA | NA |
| eICU | CRRT | class1 | OR 1.12; 95% CI 0.71–1.71; p = 0.614 |  |
| eICU | CRRT | class3 | OR 1.07; 95% CI 0.86–1.32; p = 0.546 |  |
| eICU | CRRT | class4 | OR 2.09; 95% CI 1.60–2.72; p = <0.001 | *** |
| eICU | CRRT | class5 | OR 1.41; 95% CI 1.13–1.77; p = 0.003 | ** |
| eICU | CRRT | class6 | OR 1.35; 95% CI 1.07–1.71; p = 0.011 | * |
| eICU | CRRT | class7 | OR 1.58; 95% CI 1.10–2.25; p = 0.012 | * |
| eICU | CRRT | class8 | OR 2.17; 95% CI 1.58–2.97; p = <0.001 | *** |
| MIMIC-IV | 28day-mortality | class2 | OR 1.00; 95% CI 1.00–1.00; p = NA | NA |
| MIMIC-IV | 28day-mortality | class1 | OR 1.96; 95% CI 1.36–2.80; p = <0.001 | *** |
| MIMIC-IV | 28day-mortality | class3 | OR 1.18; 95% CI 1.00–1.41; p = 0.057 |  |
| MIMIC-IV | 28day-mortality | class4 | OR 4.10; 95% CI 3.21–5.24; p = <0.001 | *** |
| MIMIC-IV | 28day-mortality | class5 | OR 1.70; 95% CI 1.39–2.07; p = <0.001 | *** |
| MIMIC-IV | 28day-mortality | class6 | OR 1.56; 95% CI 1.28–1.91; p = <0.001 | *** |
| MIMIC-IV | 28day-mortality | class7 | OR 2.55; 95% CI 1.77–3.64; p = <0.001 | *** |
| MIMIC-IV | 28day-mortality | class8 | OR 2.12; 95% CI 1.49–2.98; p = <0.001 | *** |
| eICU | 28day-mortality | class2 | OR 1.00; 95% CI 1.00–1.00; p = NA | NA |
| eICU | 28day-mortality | class1 | OR 2.13; 95% CI 1.43–3.11; p = <0.001 | *** |
| eICU | 28day-mortality | class3 | OR 1.30; 95% CI 1.05–1.61; p = 0.017 | * |
| eICU | 28day-mortality | class4 | OR 3.47; 95% CI 2.69–4.48; p = <0.001 | *** |
| eICU | 28day-mortality | class5 | OR 1.80; 95% CI 1.44–2.26; p = <0.001 | *** |
| eICU | 28day-mortality | class6 | OR 2.07; 95% CI 1.66–2.60; p = <0.001 | *** |
| eICU | 28day-mortality | class7 | OR 2.85; 95% CI 2.05–3.94; p = <0.001 | *** |
| eICU | 28day-mortality | class8 | OR 2.31; 95% CI 1.68–3.17; p = <0.001 | *** |

*including: age、sex、race、weight、icu-type.

Abbreviation: CRRT, Continuous renal replacement therapy; MV, Mechanical ventilation;

**Table S6: Univariable Cox Regression Analysis between WBC trajectory and 28day-mortality**

| Database | Class | HR(95%CI; p value) | Significance |
| --- | --- | --- | --- |
| MIMIC-IV | class2 | HR 1.00; 95% CI 1.00-1.00; p = NA |  |
| MIMIC-IV | class1 | HR 1.68; 95% CI 1.24-2.27; p = <0.001 | *** |
| MIMIC-IV | class3 | HR 1.12; 95% CI 0.97-1.31; p = 0.127 |  |
| MIMIC-IV | class4 | HR 3.00; 95% CI 2.48-3.62; p = <0.001 | *** |
| MIMIC-IV | class5 | HR 1.44; 95% CI 1.21-1.71; p = <0.001 | *** |
| MIMIC-IV | class6 | HR 1.40; 95% CI 1.18-1.66; p = <0.001 | *** |
| MIMIC-IV | class7 | HR 2.08; 95% CI 1.56-2.77; p = <0.001 | *** |
| MIMIC-IV | class8 | HR 1.80; 95% CI 1.35-2.39; p = <0.001 | *** |
| eICU | class2 | HR 1.00; 95% CI 1.00-1.00; p = NA |  |
| eICU | class1 | HR 1.90; 95% CI 1.34-2.69; p = <0.001 | *** |
| eICU | class3 | HR 1.32; 95% CI 1.08-1.61; p = 0.007 | ** |
| eICU | class4 | HR 3.03; 95% CI 2.42-3.80; p = <0.001 | *** |
| eICU | class5 | HR 1.71; 95% CI 1.39-2.10; p = <0.001 | *** |
| eICU | class6 | HR 1.94; 95% CI 1.58-2.39; p = <0.001 | *** |
| eICU | class7 | HR 2.60; 95% CI 1.95-3.46; p =<0.001 | *** |
| eICU | class8 | HR 2.12; 95% CI 1.60-2.82; p = <0.001 | *** |

**Table S7: Univariable Logistic Regression Analysis between WBC trajectory and secondary outcomes**

| Database | Variable | Class | OR (95% CI; p value) | Significance |
| --- | --- | --- | --- | --- |
| MIMIC-IV | Vasopressors | class2 | OR 1.00; 95% CI 1.00–1.00; p = NA | NA |
| MIMIC-IV | Vasopressors | class1 | OR 0.79; 95% CI 0.58–1.07; p = 0.128 |  |
| MIMIC-IV | Vasopressors | class3 | OR 1.16; 95% CI 1.02–1.32; p = 0.025 | * |
| MIMIC-IV | Vasopressors | class4 | OR 1.81; 95% CI 1.44–2.27; p = <0.001 | *** |
| MIMIC-IV | Vasopressors | class5 | OR 1.97; 95% CI 1.67–2.33; p = <0.001 | *** |
| MIMIC-IV | Vasopressors | class6 | OR 1.63; 95% CI 1.39–1.92; p = <0.001 | *** |
| MIMIC-IV | Vasopressors | class7 | OR 2.32; 95% CI 1.64–3.33; p = <0.001 | *** |
| MIMIC-IV | Vasopressors | class8 | OR 2.72; 95% CI 1.94–3.87; p = <0.001 | *** |
| eICU | Vasopressors | class2 | OR 1.00; 95% CI 1.00–1.00; p = NA | NA |
| eICU | Vasopressors | class1 | OR 1.32; 95% CI 0.97–1.78; p = 0.075 |  |
| eICU | Vasopressors | class3 | OR 1.06; 95% CI 0.91–1.23; p = 0.449 |  |
| eICU | Vasopressors | class4 | OR 2.38; 95% CI 1.95–2.92; p = <0.001 | *** |
| eICU | Vasopressors | class5 | OR 1.62; 95% CI 1.38–1.90; p = <0.001 | *** |
| eICU | Vasopressors | class6 | OR 1.58; 95% CI 1.34–1.87; p = <0.001 | *** |
| eICU | Vasopressors | class7 | OR 2.97; 95% CI 2.28–3.88; p = <0.001 | *** |
| eICU | Vasopressors | class8 | OR 3.48; 95% CI 2.72–4.46; p = <0.001 | *** |
| MIMIC-IV | Invasive.MV | class2 | OR 1.00; 95% CI 1.00–1.00; p = NA | NA |
| MIMIC-IV | Invasive.MV | class1 | OR 0.56; 95% CI 0.40–0.79; p = <0.001 | *** |
| MIMIC-IV | Invasive.MV | class3 | OR 1.31; 95% CI 1.11–1.54; p = 0.001 | ** |
| MIMIC-IV | Invasive.MV | class4 | OR 1.66; 95% CI 1.24–2.26; p = <0.001 | *** |
| MIMIC-IV | Invasive.MV | class5 | OR 1.43; 95% CI 1.17–1.76; p = <0.001 | *** |
| MIMIC-IV | Invasive.MV | class6 | OR 1.46; 95% CI 1.19–1.79; p = <0.001 | *** |
| MIMIC-IV | Invasive.MV | class7 | OR 2.18; 95% CI 1.36–3.68; p = 0.002 | ** |
| MIMIC-IV | Invasive.MV | class8 | OR 2.41; 95% CI 1.53–4.00; p = <0.001 | *** |
| eICU | Invasive.MV | class2 | OR 1.00; 95% CI 1.00–1.00; p = NA | NA |
| eICU | Invasive.MV | class1 | OR 0.57; 95% CI 0.42–0.77; p = <0.001 | *** |
| eICU | Invasive.MV | class3 | OR 1.18; 95% CI 1.00–1.38; p = 0.053 |  |
| eICU | Invasive.MV | class4 | OR 1.31; 95% CI 1.03–1.67; p = 0.032 | * |
| eICU | Invasive.MV | class5 | OR 1.16; 95% CI 0.97–1.39; p = 0.108 |  |
| eICU | Invasive.MV | class6 | OR 1.36; 95% CI 1.12–1.65; p = 0.002 | ** |
| eICU | Invasive.MV | class7 | OR 1.59; 95% CI 1.14–2.26; p = 0.008 | ** |
| eICU | Invasive.MV | class8 | OR 1.80; 95% CI 1.31–2.52; p = <0.001 | *** |
| MIMIC-IV | CRRT | class2 | OR 1.00; 95% CI 1.00–1.00; p = NA | NA |
| MIMIC-IV | CRRT | class1 | OR 1.10; 95% CI 0.56–1.99; p = 0.755 |  |
| MIMIC-IV | CRRT | class3 | OR 1.05; 95% CI 0.80–1.38; p = 0.737 |  |
| MIMIC-IV | CRRT | class4 | OR 7.35; 95% CI 5.46–9.95; p = <0.001 | *** |
| MIMIC-IV | CRRT | class5 | OR 2.08; 95% CI 1.57–2.78; p = <0.001 | *** |
| MIMIC-IV | CRRT | class6 | OR 2.54; 95% CI 1.93–3.36; p = <0.001 | *** |
| MIMIC-IV | CRRT | class7 | OR 5.50; 95% CI 3.65–8.23; p = <0.001 | *** |
| MIMIC-IV | CRRT | class8 | OR 4.98; 95% CI 3.34–7.35; p = <0.001 | *** |
| eICU | CRRT | class2 | OR 1.00; 95% CI 1.00–1.00; p = NA | NA |
| eICU | CRRT | class1 | OR 1.09; 95% CI 0.69–1.66; p = 0.696 |  |
| eICU | CRRT | class3 | OR 1.07; 95% CI 0.87–1.33; p = 0.507 |  |
| eICU | CRRT | class4 | OR 2.02; 95% CI 1.55–2.62; p = <0.001 | *** |
| eICU | CRRT | class5 | OR 1.39; 95% CI 1.11–1.74; p = 0.004 | ** |
| eICU | CRRT | class6 | OR 1.33; 95% CI 1.05–1.67; p = 0.017 | * |
| eICU | CRRT | class7 | OR 1.54; 95% CI 1.07–2.18; p = 0.018 | * |
| eICU | CRRT | class8 | OR 2.02; 95% CI 1.48–2.75; p = <0.001 | *** |
| MIMIC-IV | 28day-mortality | class2 | OR 1.00; 95% CI 1.00–1.00; p = NA | NA |
| MIMIC-IV | 28day-mortality | class1 | OR 1.80; 95% CI 1.26–2.54; p = 0.001 | ** |
| MIMIC-IV | 28day-mortality | class3 | OR 1.13; 95% CI 0.96–1.33; p = 0.158 |  |
| MIMIC-IV | 28day-mortality | class4 | OR 3.67; 95% CI 2.91–4.64; p = <0.001 | *** |
| MIMIC-IV | 28day-mortality | class5 | OR 1.48; 95% CI 1.22–1.79; p = <0.001 | *** |
| MIMIC-IV | 28day-mortality | class6 | OR 1.44; 95% CI 1.19–1.75; p = <0.001 | *** |
| MIMIC-IV | 28day-mortality | class7 | OR 2.27; 95% CI 1.60–3.19; p = <0.001 | *** |
| MIMIC-IV | 28day-mortality | class8 | OR 1.95; 95% CI 1.39–2.71; p = <0.001 | *** |
| eICU | 28day-mortality | class2 | OR 1.00; 95% CI 1.00–1.00; p = NA | NA |
| eICU | 28day-mortality | class1 | OR 1.98; 95% CI 1.34–2.88; p = <0.001 | *** |
| eICU | 28day-mortality | class3 | OR 1.34; 95% CI 1.08–1.66; p = 0.007 | ** |
| eICU | 28day-mortality | class4 | OR 3.37; 95% CI 2.62–4.34; p = <0.001 | *** |
| eICU | 28day-mortality | class5 | OR 1.77; 95% CI 1.42–2.22; p = <0.001 | *** |
| eICU | 28day-mortality | class6 | OR 2.06; 95% CI 1.65–2.58; p = <0.001 | *** |
| eICU | 28day-mortality | class7 | OR 2.81; 95% CI 2.03–3.86; p = <0.001 | *** |
| eICU | 28day-mortality | class8 | OR 2.27; 95% CI 1.65–3.10; p = <0.001 | *** |

Abbreviation: CRRT, Continuous renal replacement therapy; MV, Mechanical ventilation;

**Table S8: Multivariable Cox Regression Analysis between WBC trajectory and 28day-mortality adjusted by all variables**

| **Database** | **CLASS** | **HR(95%CI; p value)** | **Significance** |
| --- | --- | --- | --- |
| MIMIC-IV | class2 | HR 1.00; 95% CI 1.00-1.00; p = NA |  |
| MIMIC-IV | class1 | HR 1.35; 95% CI 0.99-1.84; p = 0.059 |  |
| MIMIC-IV | class3 | HR 1.22; 95% CI 1.04-1.44; p = 0.015 | * |
| MIMIC-IV | class4 | HR 2.21; 95% CI 1.70-2.87; p = <0.001 | *** |
| MIMIC-IV | class 5 | HR 1.44; 95% CI 1.17-1.77; p = <0.001 | *** |
| MIMIC-IV | class6 | HR 1.30; 95% CI 1.07-1.59; p = 0.010 | ** |
| MIMIC-IV | class7 | HR 1.70; 95% CI 1.19-2.42; p = 0.004 | ** |
| MIMIC-IV | class8 | HR 1.48; 95% CI 1.05-2.09; p = 0.025 | * |
| eICU | class2 | HR 1.00; 95% CI 1.00-1.00; p = NA |  |
| eICU | class1 | HR 1.57; 95% CI 1.09-2.25; p = 0.014 | * |
| eICU | class3 | HR 1.33; 95% CI 1.08-1.64; p = 0.007 | ** |
| eICU | class4 | HR 2.41; 95% CI 1.77-3.29; p = <0.001 | *** |
| eICU | class5 | HR 1.71; 95% CI 1.34-2.17; p = <0.001 | *** |
| eICU | class6 | HR 1.74; 95% CI 1.38-2.20; p = <0.001 | *** |
| eICU | class7 | HR 2.26; 95% CI 1.57-3.25; p = <0.001 | *** |
| eICU | class8 | HR. 1.59; 95% CI 1.11-2.27; p = 0.011 | * |

**Table S9: Multivariable Logistic Regression Analysis between WBC trajectory and secondary outcomes by all variables**

| Database | Variable | Class | OR (95% CI; p value) | Significance |
| --- | --- | --- | --- | --- |
| MIMIC-IV | Vasopressors | class2 | OR 1.00; 95% CI 1.00–1.00; p = NA | NA |
| MIMIC-IV | Vasopressors | Class1 | 0R 0.63; 95% CI 0.42-0.93; p = 0.019 | * |
| MIMIC-IV | Vasopressors | Class3 | 0R 1.06; 95% CI 0.89-1.26; p = 0.500 |  |
| MIMIC-IV | Vasopressors | Class4 | 0R 0.77; 95% CI 0.53-1.12; p = 0.172 |  |
| MIMIC-IV | Vasopressors | class5 | 0R 1.28; 95% CI 1.00-1.65; p = 0.051 |  |
| MIMIC-IV | Vasopressors | Class6 | OR 1.12; 95% CI 0.88-1.42; p = 0.371 |  |
| MIMIC-IV | Vasopressors | Class7 | OR 0.87; 95% CI 0.53-1.45; p = 0.595 |  |
| MIMIC-IV | Vasopressors | Class8 | OR 1.07; 95% CI 0.65-1.77; p = 0.791 |  |
| eICU | Vasopressors | class2 | OR 1.00; 95% CI 1.00-1.00; p = NA | NA |
| eICU | Vasopressors | Class1 | OR 0.95; 95% CI 0.67-1.33; p = 0.769 |  |
| eICU | Vasopressors | Class3 | OR 1.00; 95% CI 0.84-1.19; p = 0.987 |  |
| eICU | Vasopressors | Class4 | OR 1.25; 95% CI 0.92-1.70; p = 0.160 |  |
| eICU | Vasopressors | class5 | OR 1.26; 95% CI 1.02-1.57; p = 0.031 | * |
| eICU | Vasopressors | Class6 | OR 1.14; 95% CI 0.93-1.41; p = 0.207 |  |
| eICU | Vasopressors | Class7 | OR 1.66; 95% CI 1.15-2.40; p = 0.007 | ** |
| eICU | Vasopressors | Class8 | OR 1.62; 95% CI 1.15-2.29; p = 0.006 | ** |
| MIMIC-IV | Invasive.MV | class2 | OR 1.00; 95% CI 1.00-1.00; p = NA | NA |
| MIMIC-IV | Invasive.MV | Class1 | OR 0.48; 95% CI 0.31-0.74; p = <0.001 | *** |
| MIMIC-IV | Invasive.MV | Class3 | OR 1.33; 95% CI 1.07-1.65; p = 0.010 | * |
| MIMIC-IV | Invasive.MV | Class4 | OR 1.76; 95% CI 1.09-2.88; p = 0.023 | * |
| MIMIC-IV | Invasive.MV | class5 | OR 1.11; 95% CI 0.81-1.52; p = 0.526 |  |
| MIMIC-IV | Invasive.MV | Class6 | OR 1.48; 95% CI 1.09-2.00; p = 0.011 | * |
| MIMIC-IV | Invasive.MV | Class7 | OR 1.65; 95% CI 0.86-3.30; p = 0.144 |  |
| MIMIC-IV | Invasive.MV | Class8 | OR 2.16; 95% CI 1.13-4.25; p = 0.023 | * |
| eICU | Invasive.MV | class2 | OR 1.00; 95% CI 1.00-1.00; p = NA | NA |
| eICU | Invasive.MV | Class1 | OR 0.53; 95% CI 0.36-0.79; p = 0.002 | ** |
| eICU | Invasive.MV | Class3 | OR 1.16; 95% CI 0.94-1.43; p = 0.176 |  |
| eICU | Invasive.MV | Class4 | OR 0.90; 95% CI 0.60-1.36; p = 0.620 |  |
| eICU | Invasive.MV | class5 | OR 0.94; 95% CI 0.71-1.23; p = 0.634 |  |
| eICU | Invasive.MV | Class6 | OR 1.11; 95% CI 0.85-1.46; p = 0.439 |  |
| eICU | Invasive.MV | Class7 | OR 1.17; 95% CI 0.71-1.95; p = 0.546 |  |
| eICU | Invasive.MV | Class8 | OR 1.26; 95% CI 0.78-2.05; p = 0.354 |  |
| MIMIC-IV | CRRT | class2 | OR 1.00; 95% CI 1.00-1.00; p = NA | NA |
| MIMIC-IV | CRRT | Class1 | OR 0.86; 95% CI 0.37-1.86; p = 0.708 |  |
| MIMIC-IV | CRRT | Class3 | OR 1.18; 95% CI 0.83-1.69; p = 0.366 |  |
| MIMIC-IV | CRRT | Class4 | OR 3.08; 95% CI 1.79-5.31; p = <0.001 | *** |
| MIMIC-IV | CRRT | class5 | OR 1.45; 95% CI 0.95-2.23; p = 0.088l |  |
| MIMIC-IV | CRRT | Class6 | OR 2.31; 95% CI 1.54-3.49; p = <0.001 | *** |
| MIMIC-IV | CRRT | Class7 | OR 2.63; 95% CI 1.32-5.23; p = 0.006 | ** |
| MIMIC-IV | CRRT | Class8 | OR 2.45; 95% CI 1.27-4.69; p = 0.007 | ** |
| eICU | CRRT | class2 | OR 1.00; 95% CI 1.00-1.00; p = NA | NA |
| eICU | CRRT | Class1 | OR 1.04; 95% CI 0.59-1.78; p = 0.898 |  |
| eICU | CRRT | Class3 | OR 0.93; 95% CI 0.70-1.23; p = 0.592 |  |
| eICU | CRRT | Class4 | OR 1.04; 95% CI 0.64-1.66; p = 0.880 |  |
| eICU | CRRT | class5 | OR 1.13; 95% CI 0.80-1.59; p = 0.498 |  |
| eICU | CRRT | Class6 | OR 1.01; 95% CI 0.72-1.42; p = 0.939 |  |
| eICU | CRRT | Class7 | OR 0.73; 95% CI 0.40-1.30; p = 0.293 |  |
| eICU | CRRT | Class8 | OR 0.99; 95% CI 0.58-1.65; p =0.958 |  |
| MIMIC-IV | 28day-mortality | class2 | OR 1.00; 95% CI 1.00-1.00; p = NA | NA |
| MIMIC-IV | 28day-mortality | Class1 | OR 1.42; 95% CI 0.95-2.09; p = 0.079 |  |
| MIMIC-IV | 28day-mortality | Class3 | OR 1.29; 95% CI 1.06-1.57; p = 0.010 | ** |
| MIMIC-IV | 28day-mortality | Class4 | OR 2.84; 95% CI 2.00-4.04; p = <0.001 | *** |
| MIMIC-IV | 28day-mortality | class5 | OR 1.54; 95% CI 1.20-2.00; p = <0.001 | *** |
| MIMIC-IV | 28day-mortality | Class6 | OR 1.39; 95%CI 1.08-1.78; p = 0.009 | ** |
| MIMIC-IV | 28day-mortality | Class7 | OR 1.93; 95% CI 1.21-3.05; p = 0.005 | ** |
| MIMIC-IV | 28day-mortality | Class8 | OR 1.44; 95% CI 0.92-2.25; p = 0.112 |  |
| eICU | 28day-mortality | class2 | OR 1.00; 95% CI 1.00-1.00; p = NA | NA |
| eICU | 28day-mortality | Class1 | OR 1.61; 95% CI 1.05-2.44; p = 0.027 | * |
| eICU | 28day-mortality | Class3 | OR 1.36; 95% CI 1.08-1.72; p = 0.010 | * |
| eICU | 28day-mortality | Class4 | OR 2.73; 95% CI 1.89-3.93; p = <0.001 | *** |
| eICU | 28day-mortality | class5 | OR 1.78; 95% CI 1.36-2.35; p = <0.001 | *** |
| eICU | 28day-mortality | Class6 | OR 1.84; 95% CI 1.41-2.41; p = <0.001 | *** |
| eICU | 28day-mortality | Class7 | OR 2.52; 95% CI 1.63-3.90; p = <0.001 | *** |
| eICU | 28day-mortality | Class8 | OR 1.65; 95% CI 1.08-2.49; p = 0.019 | * |

Abbreviation: CRRT, Continuous renal replacement therapy; MV, Mechanical ventilation;

**Table S10: Baseline characteristics by WBC trajectory in MIMIC-IV**

| MIMIC-IV | Class | 1 (N=182) | 2 (N=1382) | 3 (N=2710) | 4 (N=438) | 5 (N=1158) | 6 (N=1168) | 7 (N=173) | 8 (N=199) | p |
| --- | --- | --- | --- | --- | --- | --- | --- | --- | --- | --- |
| Age |  | 62.4 (55.0 to 71.0) | 66.4 (54.9 to 77.6) | 68.4 (55.7 to 78.8) | 64.1 (54.4 to 76.3) | 65.3 (53.8 to 75.6) | 68.6 (56.6 to 78.1) | 64.2 (51.1 to 75.3) | 66.1 (53.1 to 75.6) | <.001 |
| Gender | Male | 111 (61%) | 889 (64.3%) | 1555 (57.4%) | 237 (54.1%) | 694 (59.9%) | 616 (52.7%) | 91 (52.6%) | 94 (47.2%) | <.001 |
|  | Female | 71 (39%) | 493 (35.7%) | 1155 (42.6%) | 201 (45.9%) | 464 (40.1%) | 552 (47.3%) | 82 (47.4%) | 105 (52.8%) |  |
| ICU type | SICU | 101 (55.5%) | 618 (44.7%) | 1131 (41.7%) | 210 (47.9%) | 481 (41.5%) | 495 (42.4%) | 79 (45.7%) | 93 (46.7%) | <.001 |
|  | CCU | 15 (8.2%) | 278 (20.1%) | 785 (29%) | 46 (10.5%) | 311 (26.9%) | 344 (29.5%) | 24 (13.9%) | 31 (15.6%) |  |
|  | OTHER | 66 (36.3%) | 486 (35.2%) | 794 (29.3%) | 182 (41.6%) | 366 (31.6%) | 329 (28.2%) | 70 (40.5%) | 75 (37.7%) |  |
| Race | WHITE | 112 (61.5%) | 860 (62.2%) | 1664 (61.4%) | 243 (55.5%) | 736 (63.6%) | 747 (64%) | 109 (63%) | 131 (65.8%) | .035 |
|  | ASIAN | 5 (2.7%) | 40 (2.9%) | 74 (2.7%) | 13 (3%) | 27 (2.3%) | 28 (2.4%) | 1 (0.6%) | 4 (2%) |  |
|  | BLACK | 16 (8.8%) | 113 (8.2%) | 225 (8.3%) | 50 (11.4%) | 73 (6.3%) | 101 (8.6%) | 11 (6.4%) | 15 (7.5%) |  |
|  | LATINO | 8 (4.4%) | 39 (2.8%) | 66 (2.4%) | 22 (5%) | 30 (2.6%) | 45 (3.9%) | 7 (4%) | 4 (2%) |  |
|  | OTHER | 41 (22.5%) | 330 (23.9%) | 681 (25.1%) | 110 (25.1%) | 292 (25.2%) | 247 (21.1%) | 45 (26%) | 45 (22.6%) |  |
| Weight |  | 75.0 (65.5 to 90.0) | 79.2 (67.2 to 96.0) | 81.3 (67.2 to 98.3) | 83.2 (69.1 to 100.0) | 83.0 (70.0 to 100.0) | 79.0 (65.8 to 95.0) | 85.0 (70.0 to 106.0) | 78.9 (68.2 to 97.2) | <.001 |
| SOFA |  | 9.0 (6.0 to 12.0) | 8.0 (5.0 to 10.0) | 7.0 (5.0 to 10.0) | 10.0 (7.0 to 14.0) | 8.0 (6.0 to 11.0) | 8.0 (6.0 to 11.0) | 10.0 (8.0 to 13.0) | 11.0 (7.0 to 13.0) | <.001 |
| Comorbidity |  |  |  |  |  |  |  |  |  |  |
|  | CHF | 48 (26.4%) | 421 (30.5%) | 961 (35.5%) | 117 (26.7%) | 426 (36.8%) | 408 (34.9%) | 59 (34.1%) | 58 (29.1%) | <.001 |
|  | MI | 17 (9.3%) | 229 (16.6%) | 502 (18.5%) | 69 (15.8%) | 274 (23.7%) | 230 (19.7%) | 27 (15.6%) | 31 (15.6%) | <.001 |
|  | CPD | 48 (26.4%) | 369 (26.7%) | 812 (30%) | 117 (26.7%) | 351 (30.3%) | 344 (29.5%) | 47 (27.2%) | 53 (26.6%) | .301 |
|  | CKD | 38 (20.9%) | 306 (22.1%) | 586 (21.6%) | 115 (26.3%) | 262 (22.6%) | 294 (25.2%) | 43 (24.9%) | 39 (19.6%) | .132 |
|  | Rheumatism | 12 (6.6%) | 39 (2.8%) | 103 (3.8%) | 25 (5.7%) | 43 (3.7%) | 43 (3.7%) | 9 (5.2%) | 12 (6%) | .033 |
|  | Liver disease | 68 (37.4%) | 323 (23.4%) | 401 (14.8%) | 137 (31.3%) | 232 (20%) | 187 (16%) | 56 (32.4%) | 47 (23.6%) | <.001 |
|  | Diabetes | 39 (21.4%) | 414 (30%) | 891 (32.9%) | 127 (29%) | 354 (30.6%) | 355 (30.4%) | 54 (31.2%) | 50 (25.1%) | .016 |
|  | Cancer | 67 (36.8%) | 166 (12%) | 252 (9.3%) | 69 (15.8%) | 128 (11.1%) | 145 (12.4%) | 20 (11.6%) | 32 (16.1%) | <.001 |
| Vital signs |  |  |  |  |  |  |  |  |  |  |
|  | Temperature | 38.1 (37.1 to 38.9) | 37.9 (36.3 to 38.7) | 37.9 (36.3 to 38.7) | 37.8 (35.9 to 38.8) | 37.9 (37.0 to 38.7) | 37.8 (35.8 to 38.6) | 37.6 (35.6 to 38.6) | 37.8 (35.5 to 38.8) | .004 |
|  | HR | 122.0 (107.0 to 141.0) | 116.0 (63.0 to 131.0) | 118.0 (101.0 to 133.0) | 125.5 (111.0 to 142.0) | 122.0 (108.0 to 137.0) | 122.0 (107.0 to 136.0) | 126.0 (111.0 to 138.0) | 129.0 (115.0 to 142.0) | <.001 |
|  | RR | 33.0 (29.0 to 37.5) | 32.0 (28.0 to 37.0) | 32.0 (28.0 to 37.0) | 34.0 (30.0 to 39.0) | 33.0 (29.0 to 38.0) | 33.0 (29.0 to 38.0) | 33.0 (28.0 to 37.5) | 35.0 (32.0 to 40.0) | <.001 |
|  | MAP | 52.0 (46.0 to 59.0) | 53.0 (46.0 to 58.0) | 53.0 (46.0 to 59.0) | 51.0 (42.0 to 57.0) | 51.0 (43.0 to 57.0) | 52.0 (44.0 to 58.0) | 51.0 (42.0 to 56.0) | 49.0 (37.0 to 55.0) | <.001 |
|  | SpO2 | 89.0 (86.0 to 92.0) | 90.0 (86.0 to 92.0) | 90.0 (86.0 to 92.0) | 89.0 (83.0 to 91.0) | 89.0 (85.0 to 92.0) | 89.0 (85.0 to 92.0) | 89.0 (85.0 to 92.0) | 89.0 (83.5 to 91.0) | <.001 |
| Laboratory |  |  |  |  |  |  |  |  |  |  |
|  | Hemoglobin | 7.6 (6.7 to 8.8) | 8.6 (7.5 to 10.0) | 8.8 (7.7 to 10.3) | 8.0 (7.0 to 9.5) | 8.5 (7.4 to 10.0) | 8.4 (7.4 to 9.9) | 8.1 (7.0 to 9.6) | 8.5 (7.3 to 9.6) | <.001 |
|  | WBC | 2.0 (1.2 to 2.7) | 9.3 (4.6 to 11.1) | 14.4 (12.6 to 16.8) | 29.1 (23.0 to 36.8) | 21.7 (18.1 to 25.8) | 19.6 (17.0 to 23.4) | 33.1 (25.3 to 41.0) | 30.6 (26.1 to 37.5) | <.001 |
|  | Plt | 47.5 (23.0 to 87.0) | 112.5 (72.0 to 160.0) | 141.0 (93.0 to 197.0) | 127.5 (68.0 to 219.0) | 135.5 (87.0 to 204.0) | 140.0 (85.0 to 213.5) | 121.0 (63.0 to 182.0) | 128.0 (62.5 to 186.0) | <.001 |
|  | Albumin | 2.7 (2.3 to 3.1) | 2.9 (2.4 to 3.2) | 2.9 (2.4 to 3.3) | 2.4 (2.0 to 2.9) | 2.7 (2.3 to 3.1) | 2.7 (2.2 to 3.2) | 2.4 (2.1 to 2.9) | 2.3 (2.0 to 2.8) | <.001 |
|  | ALT | 35.5 (19.0 to 80.0) | 34.0 (19.0 to 75.0) | 31.0 (18.0 to 75.0) | 49.5 (24.0 to 150.0) | 41.0 (22.0 to 122.0) | 33.0 (18.0 to 79.5) | 44.0 (21.0 to 142.0) | 45.0 (25.0 to 130.5) | <.001 |
|  | AST | 55.5 (31.0 to 120.0) | 57.0 (30.0 to 131.0) | 48.0 (28.0 to 113.0) | 96.0 (43.0 to 289.0) | 69.0 (33.0 to 221.0) | 54.0 (27.0 to 143.5) | 72.0 (32.0 to 231.0) | 84.0 (41.0 to 270.0) | <.001 |
|  | Bicarbonate | 20.0 (17.0 to 28.0) | 22.0 (18.0 to 30.0) | 22.0 (18.0 to 30.0) | 17.0 (13.0 to 22.0) | 20.0 (16.0 to 29.0) | 20.0 (17.0 to 29.0) | 17.0 (14.0 to 21.0) | 17.0 (13.0 to 21.0) | <.001 |
|  | Bun | 30.0 (18.0 to 54.0) | 28.0 (18.0 to 48.0) | 30.0 (19.0 to 48.0) | 48.0 (28.0 to 75.0) | 35.0 (22.0 to 54.0) | 35.0 (23.0 to 56.0) | 48.0 (27.0 to 70.0) | 45.0 (29.0 to 68.0) | <.001 |
|  | Cr | 1.1 (0.8 to 2.1) | 1.3 (0.9 to 2.3) | 1.3 (0.9 to 2.2) | 2.4 (1.3 to 4.3) | 1.6 (1.0 to 2.6) | 1.5 (1.0 to 2.7) | 2.0 (1.1 to 3.5) | 2.2 (1.2 to 3.3) | <.001 |
|  | Chloride | 108.5 (99.0 to 113.0) | 109.0 (98.0 to 113.0) | 109.0 (98.0 to 113.0) | 107.0 (95.0 to 113.0) | 109.0 (97.0 to 113.0) | 109.0 (97.0 to 113.0) | 109.0 (95.0 to 113.0) | 109.0 (95.0 to 114.0) | .022 |
|  | APTT | 37.3 (31.9 to 58.2) | 37.5 (31.1 to 61.7) | 36.5 (30.2 to 62.0) | 45.2 (33.7 to 78.9) | 38.4 (30.7 to 71.4) | 39.5 (31.6 to 66.4) | 41.7 (32.5 to 70.9) | 45.1 (33.5 to 83.2) | <.001 |
|  | INR | 1.4 (1.3 to 1.9) | 1.4 (1.2 to 1.8) | 1.4 (1.2 to 1.7) | 1.7 (1.4 to 2.4) | 1.5 (1.3 to 1.9) | 1.5 (1.3 to 2.0) | 1.6 (1.3 to 2.0) | 1.7 (1.4 to 2.0) | <.001 |
|  | pH | 7.4 (7.3 to 7.5) | 7.3 (7.2 to 7.5) | 7.3 (7.2 to 7.5) | 7.3 (7.2 to 7.4) | 7.3 (7.2 to 7.4) | 7.3 (7.2 to 7.5) | 7.2 (7.2 to 7.4) | 7.2 (7.1 to 7.3) | <.001 |
|  | PaO2 | 75.5 (63.0 to 95.0) | 75.0 (63.0 to 96.0) | 74.0 (63.0 to 93.0) | 69.0 (60.0 to 82.0) | 72.0 (61.6 to 89.0) | 72.0 (62.0 to 87.0) | 70.0 (61.0 to 84.0) | 69.0 (60.5 to 80.0) | <.001 |
|  | PaO2/FiO2 | 159.0 (108.0 to 258.0) | 168.0 (106.7 to 248.0) | 155.9 (100.0 to 231.2) | 127.2 (83.0 to 200.0) | 137.5 (87.1 to 207.5) | 138.0 (90.0 to 219.5) | 130.0 (87.5 to 210.0) | 120.0 (81.2 to 176.5) | <.001 |
|  | PaCO2 | 44.0 (36.0 to 52.0) | 47.0 (39.0 to 55.0) | 47.0 (41.0 to 55.0) | 47.0 (40.0 to 54.0) | 48.0 (41.0 to 57.0) | 47.0 (40.5 to 54.0) | 48.0 (42.0 to 57.0) | 49.0 (42.0 to 57.5) | <.001 |
|  | Sodium | 135.0 (132.0 to 140.0) | 136.0 (133.0 to 139.0) | 135.0 (133.0 to 139.0) | 135.0 (132.0 to 138.0) | 135.0 (132.0 to 139.0) | 135.0 (132.0 to 139.0) | 134.0 (131.0 to 141.0) | 135.0 (131.0 to 139.0) | <.001 |
|  | Potassium | 3.5 (3.2 to 4.0) | 3.6 (3.3 to 4.2) | 3.6 (3.3 to 4.2) | 3.6 (3.3 to 4.4) | 3.6 (3.3 to 4.6) | 3.6 (3.3 to 4.3) | 3.7 (3.3 to 4.6) | 3.6 (3.3 to 4.2) | .313 |
|  | Lactate | 1.9 (1.3 to 3.3) | 1.9 (1.3 to 3.2) | 2.1 (1.4 to 3.4) | 3.0 (1.9 to 5.4) | 2.5 (1.6 to 4.2) | 2.5 (1.6 to 4.4) | 2.9 (1.9 to 5.7) | 3.8 (2.5 to 5.6) | <.001 |

Data are presented as count (percent) or median (interquartile range [IQR])

Abbreviation: CCU, Coronary care unit ; CHF, Chronic heart failure; CKD, Chronic kidney disease; SICU, Surgery intensive care unit; SOFA, Sequential Organ Failure Assessment; WBC, white blood cell.

**Table S11: Baseline characteristics by WBC trajectory in eICU**

| eICU | Class | 1 (N=213) | 2 (N=1320) | 3 (N=2176) | 4 (N=571) | 5 (N=1354) | 6 (N=1201) | 7 (N=277) | 8 (N=335) | p |
| --- | --- | --- | --- | --- | --- | --- | --- | --- | --- | --- |
| Age |  | 60.0 (53.0 to 71.0) | 65.0 (54.0 to 75.0) | 67.0 (55.0 to 77.0) | 66.0 (54.0 to 76.0) | 65.0 (54.0 to 76.0) | 65.0 (55.0 to 76.0) | 66.0 (55.0 to 77.0) | 67.0 (55.5 to 76.0) | .002 |
| Gender | Male | 120 (56.3%) | 762 (57.7%) | 1181 (54.3%) | 290 (50.8%) | 695 (51.3%) | 632 (52.6%) | 145 (52.3%) | 151 (45.1%) | <.001 |
|  | Female | 93 (43.7%) | 558 (42.3%) | 995 (45.7%) | 281 (49.2%) | 659 (48.7%) | 569 (47.4%) | 132 (47.7%) | 184 (54.9%) |  |
| ICU type | SICU | 152 (71.4%) | 869 (65.8%) | 1448 (66.5%) | 378 (66.2%) | 908 (67.1%) | 827 (68.9%) | 188 (67.9%) | 237 (70.7%) | .541 |
|  | CCU | 34 (16%) | 242 (18.3%) | 397 (18.2%) | 99 (17.3%) | 214 (15.8%) | 196 (16.3%) | 45 (16.2%) | 54 (16.1%) |  |
|  | OTHER | 27 (12.7%) | 209 (15.8%) | 331 (15.2%) | 94 (16.5%) | 232 (17.1%) | 178 (14.8%) | 44 (15.9%) | 44 (13.1%) |  |
| Race | WHITE | 166 (77.9%) | 1026 (77.7%) | 1659 (76.2%) | 431 (75.5%) | 1056 (78%) | 906 (75.4%) | 216 (78%) | 255 (76.1%) | .151 |
|  | ASIAN | 4 (1.9%) | 22 (1.7%) | 36 (1.7%) | 10 (1.8%) | 23 (1.7%) | 24 (2%) | 3 (1.1%) | 6 (1.8%) |  |
|  | BLACK | 22 (10.3%) | 139 (10.5%) | 244 (11.2%) | 71 (12.4%) | 123 (9.1%) | 140 (11.7%) | 29 (10.5%) | 34 (10.1%) |  |
|  | LATINO | 8 (3.8%) | 68 (5.2%) | 121 (5.6%) | 13 (2.3%) | 53 (3.9%) | 57 (4.7%) | 11 (4%) | 15 (4.5%) |  |
|  | OTHER | 13 (6.1%) | 65 (4.9%) | 116 (5.3%) | 46 (8.1%) | 99 (7.3%) | 74 (6.2%) | 18 (6.5%) | 25 (7.5%) |  |
| Weight |  | 79.5 (68.4 to 94.3) | 82.5 (67.7 to 100.0) | 81.6 (67.1 to 100.0) | 78.0 (63.5 to 95.2) | 80.5 (66.7 to 100.0) | 77.0 (63.7 to 96.4) | 77.5 (64.0 to 98.8) | 71.5 (60.0 to 90.7) | <.001 |
| SOFA |  | 9.0 (7.0 to 11.0) | 8.0 (6.0 to 10.0) | 8.0 (6.0 to 10.0) | 9.0 (7.0 to 12.0) | 8.0 (6.0 to 11.0) | 9.0 (6.0 to 11.0) | 9.0 (6.0 to 12.0) | 10.0 (8.0 to 13.0) | <.001 |
| Comorbidity |  |  |  |  |  |  |  |  |  |  |
|  | CHF | 40 (18.8%) | 342 (25.9%) | 558 (25.6%) | 101 (17.7%) | 308 (22.7%) | 257 (21.4%) | 52 (18.8%) | 55 (16.4%) | <.001 |
|  | MI | 26 (12.2%) | 201 (15.2%) | 408 (18.8%) | 87 (15.2%) | 235 (17.4%) | 214 (17.8%) | 40 (14.4%) | 50 (14.9%) | .031 |
|  | CPD | 56 (26.3%) | 434 (32.9%) | 690 (31.7%) | 162 (28.4%) | 417 (30.8%) | 385 (32.1%) | 91 (32.9%) | 87 (26%) | .113 |
|  | CKD | 43 (20.2%) | 301 (22.8%) | 494 (22.7%) | 121 (21.2%) | 287 (21.2%) | 241 (20.1%) | 61 (22%) | 72 (21.5%) | .697 |
|  | Rheumatism | 5 (2.3%) | 35 (2.7%) | 67 (3.1%) | 14 (2.5%) | 39 (2.9%) | 50 (4.2%) | 4 (1.4%) | 7 (2.1%) | .162 |
|  | Liver disease | 27 (12.7%) | 120 (9.1%) | 149 (6.8%) | 44 (7.7%) | 105 (7.8%) | 83 (6.9%) | 29 (10.5%) | 33 (9.9%) | .010 |
|  | Diabetes | 54 (25.4%) | 454 (34.4%) | 756 (34.7%) | 188 (32.9%) | 438 (32.3%) | 383 (31.9%) | 86 (31%) | 106 (31.6%) | .121 |
|  | Cancer | 92 (43.2%) | 193 (14.6%) | 292 (13.4%) | 111 (19.4%) | 172 (12.7%) | 190 (15.8%) | 41 (14.8%) | 61 (18.2%) | <.001 |
| Vital signs |  |  |  |  |  |  |  |  |  |  |
|  | Temperature | 38.2 (37.2 to 39.2) | 37.8 (35.9 to 38.6) | 37.9 (36.0 to 38.6) | 37.8 (35.8 to 38.8) | 37.9 (36.0 to 38.8) | 37.9 (35.8 to 38.7) | 37.8 (35.7 to 38.7) | 38.0 (35.7 to 38.8) | <.001 |
|  | HR | 126.0 (113.0 to 145.0) | 120.0 (102.0 to 135.0) | 122.0 (106.0 to 139.0) | 131.0 (116.0 to 146.5) | 126.0 (110.0 to 141.0) | 128.0 (113.0 to 144.0) | 130.0 (115.0 to 143.0) | 128.0 (114.0 to 144.0) | <.001 |
|  | RR | 35.0 (29.0 to 42.0) | 33.0 (28.0 to 40.0) | 34.0 (28.0 to 40.0) | 36.0 (30.0 to 42.0) | 35.0 (29.0 to 41.0) | 35.0 (30.0 to 41.0) | 35.0 (30.0 to 42.0) | 36.0 (30.0 to 42.0) | <.001 |
|  | MAP | 51.0 (44.0 to 59.0) | 53.0 (45.5 to 60.0) | 52.0 (45.0 to 60.0) | 49.0 (40.0 to 56.0) | 51.0 (43.5 to 58.0) | 51.0 (43.0 to 58.0) | 50.0 (41.0 to 56.0) | 48.0 (41.0 to 55.0) | <.001 |
|  | SpO2 | 88.0 (81.0 to 92.0) | 88.0 (82.0 to 91.0) | 87.0 (81.0 to 91.0) | 86.0 (79.0 to 91.0) | 87.0 (81.0 to 91.0) | 87.0 (80.0 to 91.0) | 87.0 (80.0 to 91.0) | 86.0 (79.0 to 90.0) | .007 |
| Laboratory |  |  |  |  |  |  |  |  |  |  |
|  | Hemoglobin | 7.6 (6.8 to 8.8) | 8.6 (7.5 to 10.1) | 8.8 (7.5 to 10.3) | 8.2 (7.2 to 9.6) | 8.6 (7.4 to 10.1) | 8.6 (7.5 to 10.1) | 8.2 (7.1 to 9.6) | 8.2 (7.1 to 9.5) | <.001 |
|  | WBC | 2.0 (1.2 to 2.7) | 9.8 (4.6 to 11.9) | 14.7 (12.8 to 17.3) | 31.7 (25.9 to 40.0) | 22.3 (18.9 to 26.4) | 20.1 (17.3 to 23.5) | 34.5 (28.2 to 41.4) | 31.4 (26.1 to 39.8) | <.001 |
|  | Plt | 45.0 (20.0 to 85.0) | 124.0 (79.0 to 168.5) | 152.0 (105.0 to 205.5) | 136.0 (74.0 to 219.0) | 151.0 (100.0 to 220.0) | 151.0 (89.0 to 220.0) | 122.0 (68.0 to 193.0) | 102.0 (47.5 to 168.0) | <.001 |
|  | Albumin | 2.1 (1.8 to 2.5) | 2.4 (2.0 to 2.8) | 2.3 (1.9 to 2.8) | 1.9 (1.5 to 2.3) | 2.2 (1.8 to 2.6) | 2.1 (1.7 to 2.6) | 2.0 (1.6 to 2.4) | 2.0 (1.6 to 2.4) | <.001 |
|  | ALT | 38.0 (22.0 to 74.0) | 30.0 (18.0 to 58.0) | 33.0 (19.0 to 64.0) | 46.0 (25.0 to 121.5) | 35.0 (20.0 to 78.0) | 37.0 (21.0 to 91.0) | 39.0 (23.0 to 93.0) | 41.0 (24.0 to 125.0) | <.001 |
|  | AST | 46.0 (30.0 to 127.0) | 39.0 (24.0 to 78.0) | 43.0 (25.0 to 94.0) | 66.0 (37.0 to 192.5) | 48.0 (27.0 to 119.0) | 51.0 (28.0 to 131.0) | 60.0 (29.0 to 161.0) | 69.0 (34.0 to 212.5) | <.001 |
|  | Bicarbonate | 21.0 (17.0 to 29.0) | 28.0 (19.0 to 32.0) | 27.0 (18.0 to 31.0) | 18.0 (15.0 to 29.0) | 21.0 (16.0 to 31.0) | 21.0 (16.0 to 31.0) | 18.0 (15.0 to 29.0) | 17.0 (14.5 to 29.5) | <.001 |
|  | Bun | 38.0 (23.0 to 64.0) | 34.0 (21.0 to 54.0) | 37.5 (24.0 to 60.0) | 53.0 (33.5 to 75.5) | 39.0 (24.0 to 60.0) | 42.0 (27.0 to 64.0) | 46.0 (31.0 to 66.0) | 48.0 (31.0 to 71.0) | <.001 |
|  | Cr | 1.5 (0.9 to 2.5) | 1.4 (0.9 to 2.6) | 1.5 (1.0 to 2.9) | 2.3 (1.3 to 4.0) | 1.8 (1.0 to 3.1) | 1.8 (1.1 to 3.2) | 1.9 (1.1 to 3.4) | 2.3 (1.5 to 3.7) | <.001 |
|  | Chloride | 110.0 (98.0 to 115.0) | 109.0 (97.0 to 114.0) | 110.0 (97.0 to 114.0) | 110.0 (96.0 to 115.0) | 110.0 (97.0 to 115.0) | 110.0 (96.0 to 115.0) | 111.0 (96.0 to 116.0) | 111.0 (96.0 to 116.0) | .162 |
|  | APTT | 38.4 (31.0 to 54.0) | 35.0 (29.7 to 48.0) | 35.6 (29.7 to 50.2) | 38.3 (33.0 to 56.7) | 36.0 (30.0 to 49.5) | 36.0 (30.0 to 51.0) | 36.8 (31.1 to 52.0) | 39.0 (32.9 to 54.3) | <.001 |
|  | INR | 1.4 (1.2 to 1.9) | 1.3 (1.1 to 1.7) | 1.3 (1.1 to 1.8) | 1.5 (1.3 to 2.1) | 1.4 (1.2 to 1.8) | 1.4 (1.2 to 1.9) | 1.5 (1.2 to 2.0) | 1.5 (1.3 to 2.0) | <.001 |
|  | pH | 7.3 (7.2 to 7.5) | 7.3 (7.2 to 7.5) | 7.3 (7.2 to 7.5) | 7.3 (7.2 to 7.4) | 7.3 (7.2 to 7.4) | 7.3 (7.2 to 7.4) | 7.3 (7.2 to 7.4) | 7.2 (7.2 to 7.4) | <.001 |
|  | PaO2 | 65.0 (54.5 to 83.2) | 67.0 (55.6 to 83.3) | 66.0 (54.7 to 82.0) | 64.0 (53.0 to 77.2) | 65.0 (54.0 to 80.0) | 65.0 (54.0 to 79.4) | 67.0 (56.2 to 84.1) | 62.0 (52.0 to 73.8) | <.001 |
|  | PaO2/FiO2 | 155.7 (98.0 to 245.0) | 150.1 (94.0 to 235.8) | 143.0 (86.5 to 219.7) | 122.6 (75.0 to 200.0) | 141.5 (84.0 to 230.0) | 130.0 (77.5 to 210.0) | 150.0 (79.0 to 225.0) | 125.7 (75.5 to 200.0) | <.001 |
|  | PaCO2 | 43.2 (36.7 to 53.0) | 48.0 (40.0 to 60.0) | 47.0 (39.0 to 58.0) | 45.0 (38.0 to 56.5) | 46.2 (38.7 to 58.1) | 47.0 (39.0 to 59.0) | 46.1 (38.0 to 57.0) | 46.0 (38.4 to 55.3) | <.001 |
|  | Sodium | 136.0 (131.0 to 146.0) | 137.0 (132.0 to 146.0) | 137.0 (132.0 to 147.0) | 135.0 (130.0 to 147.0) | 136.0 (132.0 to 147.0) | 136.0 (132.0 to 147.0) | 136.0 (131.0 to 148.0) | 135.0 (130.0 to 147.0) | .013 |
|  | Potassium | 3.3 (3.0 to 3.6) | 3.4 (3.1 to 3.8) | 3.4 (3.1 to 3.8) | 3.3 (3.0 to 3.9) | 3.3 (3.0 to 3.8) | 3.3 (3.0 to 3.8) | 3.4 (3.1 to 3.8) | 3.2 (2.9 to 3.7) | <.001 |
|  | Lactate | 2.1 (1.3 to 3.8) | 1.9 (1.2 to 3.2) | 2.1 (1.3 to 3.6) | 2.7 (1.7 to 4.9) | 2.5 (1.5 to 4.5) | 2.5 (1.5 to 4.3) | 3.0 (1.9 to 5.3) | 3.5 (2.0 to 6.0) | <.001 |
| Vasopressors |  | 76 (35.7%) | 391 (29.6%) | 671 (30.8%) | 286 (50.1%) | 548 (40.5%) | 480 (40%) | 154 (55.6%) | 199 (59.4%) | <.001 |
| Invasive.MV |  | 138 (64.8%) | 1009 (76.4%) | 1724 (79.2%) | 462 (80.9%) | 1070 (79%) | 979 (81.5%) | 232 (83.8%) | 286 (85.4%) | <.001 |
| CRRT |  | 27 (12.7%) | 155 (11.7%) | 272 (12.5%) | 121 (21.2%) | 211 (15.6%) | 180 (15%) | 47 (17%) | 71 (21.2%) | <.001 |
| 28day-mortality |  | 41 (19.2%) | 142 (10.8%) | 302 (13.9%) | 165 (28.9%) | 238 (17.6%) | 239 (19.9%) | 70 (25.3%) | 72 (21.5%) | <.001 |
| LOS of ICU |  | 7.2 (5.1 to 10.0) | 7.0 (5.4 to 10.5) | 7.1 (5.3 to 10.6) | 8.1 (5.7 to 11.9) | 7.1 (5.4 to 10.8) | 8.0 (5.7 to 12.3) | 7.1 (5.3 to 10.6) | 8.3 (6.0 to 12.9) | <.001 |
| LOS of hospital |  | 13.2 (8.2 to 21.7) | 13.0 (9.0 to 19.2) | 13.3 (9.2 to 19.8) | 14.4 (9.4 to 21.5) | 13.1 (8.9 to 20.2) | 14.5 (9.9 to 21.1) | 12.6 (8.8 to 18.7) | 14.5 (10.0 to 21.3) | <.001 |

Data are presented as count (percent) or median (interquartile range [IQR])

Abbreviation: CCU, Coronary care unit ; CHF, Chronic heart failure; CKD, Chronic kidney disease; SICU, Surgery intensive care unit; SOFA, Sequential Organ Failure Assessment; WBC, white blood cell.

**Table S12: Clinical outcomes by WBC trajectory in MIMIC-IV and eICU**

| **Database** | MIMIC-IV (N=7410) | eICU (N=7447) | p |
| --- | --- | --- | --- |
| Vasopressors | 4572 (61.7%) | 2805 (37.7%) | <.001 |
| Invasive MV | 6130 (82.7%) | 5900 (79.2%) | <.001 |
| CRRT | 797 (10.8%) | 1084 (14.6%) | <.001 |
| 28-day Mortality | 1698 (22.9%) | 1269 (17%) | <.001 |
| LOS of ICU | 7.8 (5.4 to 12.8) | 7.4 (5.5 to 11.1) | <.001 |
| LOS of hospital | 15.0 (10.0 to 23.8) | 13.6 (9.2 to 20.2) | <.001 |

Data are presented as count (percent) or median (interquartile range [IQR])

Abbreviation: CRRT, Continuous renal replacement therapy; MV, Mechanical ventilation;

**Table S13: Clinical outcomes by class in MIMIC-IV**

| MIMIC-IV by class | 1 (N=182) | 2 (N=1382) | 3 (N=2710) | 4 (N=438) | 5 (N=1158) | 6 (N=1168) | 7 (N=173) | 8 (N=199) | p |
| --- | --- | --- | --- | --- | --- | --- | --- | --- | --- |
| Vasopressors | 88 (48.4%) | 751 (54.3%) | 1572 (58%) | 299 (68.3%) | 812 (70.1%) | 771 (66%) | 127 (73.4%) | 152 (76.4%) | <.001 |
| Invasive MV | 123 (67.6%) | 1089 (78.8%) | 2247 (82.9%) | 377 (86.1%) | 975 (84.2%) | 986 (84.4%) | 154 (89%) | 179 (89.9%) | <.001 |
| CRRT | 12 (6.6%) | 83 (6%) | 170 (6.3%) | 140 (32%) | 136 (11.7%) | 163 (14%) | 45 (26%) | 48 (24.1%) | <.001 |
| 28-day Mortality | 51 (28%) | 246 (17.8%) | 532 (19.6%) | 194 (44.3%) | 281 (24.3%) | 278 (23.8%) | 57 (32.9%) | 59 (29.6%) | <.001 |
| LOS of ICU | 6.9 (5.3 to 10.9) | 7.2 (5.1 to 11.9) | 7.5 (5.3 to 12.1) | 10.0 (6.1 to 16.7) | 7.8 (5.6 to 12.7) | 8.3 (5.7 to 13.6) | 9.2 (6.2 to 14.8) | 9.6 (6.4 to 13.5) | <.001 |
| LOS of hospital | 15.9 (10.6 to 28.3) | 14.3 (9.7 to 22.9) | 14.3 (9.7 to 22.3) | 19.1 (11.1 to 29.5) | 15.5 (9.9 to 23.7) | 16.2 (10.6 to 25.4) | 17.7 (11.8 to 27.1) | 19.2 (11.9 to 25.8) | <.001 |

Data are presented as count (percent) or median (interquartile range [IQR])

Abbreviation: CRRT, Continuous renal replacement therapy; MV, Mechanical ventilation;

**Table S14:** **Baseline characteristics Grouped by Outcome (28-Day Mortality)**

|  | 28-day Mortality | Survival (N=11890) | Death (N=2967) | p |
| --- | --- | --- | --- | --- |
| Database | MIMIC-IV | 5712 (48%) | 1698 (57.2%) | <.001 |
|  | eICU | 6178 (52%) | 1269 (42.8%) |  |
| class | 1 | 303 (2.5%) | 92 (3.1%) | <.001 |
|  | 2 | 2314 (19.5%) | 388 (13.1%) |  |
|  | 3 | 4052 (34.1%) | 834 (28.1%) |  |
|  | 4 | 650 (5.5%) | 359 (12.1%) |  |
|  | 5 | 1993 (16.8%) | 519 (17.5%) |  |
|  | 6 | 1852 (15.6%) | 517 (17.4%) |  |
|  | 7 | 323 (2.7%) | 127 (4.3%) |  |
|  | 8 | 403 (3.4%) | 131 (4.4%) |  |
| Age |  | 65.0 (53.7 to 75.9) | 71.0 (60.0 to 80.9) | <.001 |
| Gender | Male | 6592 (55.4%) | 1671 (56.3%) | .401 |
|  | Female | 5298 (44.6%) | 1296 (43.7%) |  |
| ICU type | SICU | 6641 (55.9%) | 1574 (53.1%) | <.001 |
|  | CCU | 2576 (21.7%) | 539 (18.2%) |  |
|  | OTHER | 2673 (22.5%) | 854 (28.8%) |  |
| Race | WHITE | 8347 (70.2%) | 1970 (66.4%) | <.001 |
|  | ASIAN | 261 (2.2%) | 59 (2%) |  |
|  | BLACK | 1147 (9.6%) | 259 (8.7%) |  |
|  | LATINO | 464 (3.9%) | 103 (3.5%) |  |
|  | OTHER | 1671 (14.1%) | 576 (19.4%) |  |
| Weight |  | 80.8 (67.0 to 99.0) | 78.4 (65.2 to 95.3) | <.001 |
| SOFA |  | 8.0 (6.0 to 10.0) | 10.0 (7.0 to 13.0) | <.001 |
| Comorbidity |  |  |  |  |
|  | CHF | 3232 (27.2%) | 979 (33%) | <.001 |
|  | MI | 2027 (17%) | 613 (20.7%) | <.001 |
|  | CPD | 3534 (29.7%) | 929 (31.3%) | .096 |
|  | CKD | 2485 (20.9%) | 818 (27.6%) | <.001 |
|  | Rheumatism | 389 (3.3%) | 118 (4%) | .066 |
|  | Liver disease | 1418 (11.9%) | 623 (21%) | <.001 |
|  | Diabetes | 3790 (31.9%) | 959 (32.3%) | .657 |
|  | Cancer | 1463 (12.3%) | 568 (19.1%) | <.001 |
| Vital signs |  |  |  |  |
|  | Temperature | 37.9 (36.2 to 38.7) | 37.7 (35.6 to 38.6) | <.001 |
|  | HR | 122.0 (105.0 to 138.0) | 124.0 (109.0 to 140.0) | <.001 |
|  | RR | 33.0 (28.5 to 39.0) | 35.0 (30.0 to 40.0) | <.001 |
|  | MAP | 52.0 (45.0 to 59.0) | 50.0 (41.0 to 56.0) | <.001 |
|  | SpO2 | 89.0 (84.0 to 92.0) | 88.0 (81.0 to 91.0) | <.001 |
| Laboratory |  |  |  |  |
|  | Hemoglobin | 8.6 (7.5 to 10.1) | 8.4 (7.2 to 9.9) | <.001 |
|  | WBC | 16.3 (12.1 to 22.1) | 17.8 (13.1 to 24.5) | <.001 |
|  | Plt | 138.0 (87.0 to 198.0) | 120.0 (64.0 to 184.0) | <.001 |
|  | Albumin | 2.5 (2.0 to 3.0) | 2.4 (1.9 to 2.9) | <.001 |
|  | ALT | 34.0 (19.0 to 75.0) | 40.0 (21.0 to 112.0) | <.001 |
|  | AST | 48.5 (27.0 to 114.0) | 67.0 (32.0 to 205.5) | <.001 |
|  | Bicarbonate | 22.0 (17.0 to 30.0) | 19.0 (15.0 to 29.0) | <.001 |
|  | Bun | 34.0 (21.0 to 55.0) | 46.0 (29.0 to 69.0) | <.001 |
|  | Cr | 1.4 (0.9 to 2.7) | 2.0 (1.2 to 3.3) | <.001 |
|  | Chloride | 109.0 (97.0 to 114.0) | 109.0 (96.0 to 114.0) | .381 |
|  | APTT | 36.0 (30.0 to 53.0) | 42.5 (32.7 to 73.7) | <.001 |
|  | INR | 1.4 (1.2 to 1.8) | 1.6 (1.3 to 2.4) | <.001 |
|  | pH | 7.3 (7.2 to 7.5) | 7.3 (7.2 to 7.4) | <.001 |
|  | PaO2 | 70.0 (59.0 to 87.0) | 67.0 (56.0 to 81.2) | <.001 |
|  | PaO2.FiO2 | 149.0 (92.5 to 227.5) | 130.0 (79.0 to 206.7) | <.001 |
|  | PaCO2 | 47.0 (40.0 to 56.0) | 47.0 (39.8 to 58.0) | .026 |
|  | Sodium | 136.0 (132.0 to 144.0) | 136.0 (132.0 to 145.0) | .590 |
|  | Potassium | 3.5 (3.1 to 4.0) | 3.5 (3.2 to 4.3) | <.001 |
|  | Lactate | 2.2 (1.4 to 3.8) | 2.6 (1.6 to 4.7) | <.001 |
| Outcome |  |  |  |  |
|  | Vasopressors | 5615 (47.2%) | 1762 (59.4%) | <.001 |
|  | Invasive.MV | 9502 (79.9%) | 2528 (85.2%) | <.001 |
|  | CRRT | 1307 (11%) | 574 (19.3%) | <.001 |
|  | LOS of ICU | 7.4 (5.3 to 11.8) | 8.2 (6.0 to 12.0) | <.001 |
|  | LOS of hospital | 15.1 (10.2 to 23.8) | 11.2 (7.6 to 16.3) | <.001 |

Data are presented as count (percent) or median (interquartile range [IQR])

Abbreviation: CCU, Coronary care unit ; CHF, Chronic heart failure; CKD, Chronic kidney disease; SICU, Surgery intensive care unit; SOFA, Sequential Organ Failure Assessment; WBC, white blood cell.

**Table S15: Performance Metrics of the XGBoost Model on MIMIC-IV and eICU Datasets.**

|  | AUC | PPV | NPV | Accuracy | Balanced accuracy |
| --- | --- | --- | --- | --- | --- |
| Class1(MIMIC-IV) | 0.913 | 0.751 | 0.891 | 0.872 | 0.812 |
| Class1(eICU) | 0.876 | 0.741 | 0.873 | 0.861 | 0.789 |
| Class4(MIMIC-IV) | 0.923 | 0.765 | 0.897 | 0.879 | 0.823 |
| Class4(eICU) | 0.878 | 0.746 | 0.875 | 0.862 | 0.793 |
| Class7(MIMIC-IV) | 0.851 | 0.701 | 0.862 | 0.849 | 0.779 |
| Class7(eICU) | 0.829 | 0.684 | 0.851 | 0.832 | 0.767 |
| Class8(MIMIC-IV) | 0.829 | 0.678 | 0.847 | 0.828 | 0.762 |
| Class8(eICU) | 0.818 | 0.672 | 0.839 | 0.821 | 0.757 |

Abbreviation: AUC, Area Under the Curve; PPV, Positive Predictive Value; NPV, Negative Predictive Value
