# Supplementary material for: Clinical subtypes identification and feature recognition of sepsis leukocyte trajectories based on machine learning
Source: Sci Rep. 2025 Apr 10;15:12291. doi: 10.1038/s41598-025-96718-9 (PMC11986166; doi:10.1038/s41598-025-96718-9)
Supplement: Supplementary file 3 — Supplementary Material 3 [file 41598_2025_96718_MOESM3_ESM.pdf]

```
DROP AGGREGATE IF EXISTS farthest(float8, float8, float8) CASCADE;
DROP FUNCTION IF EXISTS farthest_trans(float8[], float8, float8,
float8) CASCADE;
DROP FUNCTION IF EXISTS farthest_final(float8[]) CASCADE;


CREATE OR REPLACE FUNCTION farthest_trans(
    state float8[],
    val float8,
    target_value float8,
    prefer_larger float8
)
RETURNS float8[] AS $$
DECLARE
    current_max_distance float8;
    new_distance float8;
BEGIN
    IF state IS NULL THEN
        state := ARRAY[0, 0, 0, 0, 0];
    END IF;

    current_max_distance := state[1];
    new_distance := abs(val - target_value);
    state[3] := state[3] + 1;


    IF val IS NULL THEN
        state[5] := state[5] + 1;
        RETURN state;
    END IF;


    IF state[3] = 1 AND val = target_value THEN
        state := ARRAY[new_distance, val, state[3], target_value,
state[5]];
        RETURN state;
    END IF;

    IF new_distance > current_max_distance THEN
        state := ARRAY[new_distance, val, state[3], target_value,
state[5]];
    ELSIF new_distance = current_max_distance THEN
        IF prefer_larger = 1 THEN
            IF val > state[2] THEN
                state[2] := val;
            END IF;
        ELSE
            IF val < state[2] THEN
                state[2] := val;
            END IF;
        END IF;
    END IF;
```

```
    RETURN state;
END;
$$ LANGUAGE plpgsql;


CREATE OR REPLACE FUNCTION farthest_final(
    state float8[]
)
RETURNS float8 AS $$
BEGIN
    IF state[3] = state[5] THEN
        RETURN NULL;
    ELSIF state[3] = 1 AND state[4] = state[2] THEN
        RETURN state[2];
    ELSE
        RETURN state[2];
    END IF;
END;
$$ LANGUAGE plpgsql;


CREATE AGGREGATE farthest(float8, float8, float8) (
    SFUNC = farthest_trans,
    STYPE = float8[],
    FINALFUNC = farthest_final,
    INITCOND = '{0, 0, 0, 0, 0}'
);
```
